# Lactylation‐Driven IGF2BP3‐Mediated Serine Metabolism Reprogramming and RNA m6A—Modification Promotes Lenvatinib Resistance in HCC

**DOI:** 10.1002/advs.202401399

**Published:** 2024-10-25

**Authors:** Yuanxiang Lu, Jinghan Zhu, Yuxin Zhang, Wentao Li, Yixiao Xiong, Yunhui Fan, Yang Wu, Jianping Zhao, Changzhen Shang, Huifang Liang, Wanguang Zhang

**Affiliations:** ^1^ Hepatic Surgery Center Tongji Hospital Tongji Medical College Huazhong University of Science and Technology 1095 Jiefang Avenue Wuhan Hubei 430030 China; ^2^ Department of Breast Surgery Zhengzhou University People's Hospital Henan Provincial People's Hospital Zhengzhou 450003 China; ^3^ Hubei Key Laboratory of Hepato‐Pancreato‐Biliary Diseases Wuhan Hubei 430030 China; ^4^ Department of Dermatology Tongji Hospital Tongji Medical College Huazhong University of Science and Technology Wuhan Hubei 430030 China; ^5^ Key Laboratory of Organ Transplantation Ministry of Education NHC Key Laboratory of Organ Transplantation Key Laboratory of Organ Transplantation Chinese Academy of Medical Sciences Wuhan Hubei 430030 China; ^6^ Department of Hepatobiliary Surgery Sun Yat‐sen Memorial Hospital of Sun Yat‐sen University Yanjiang West Road Guangzhou 510120 China

**Keywords:** glycolysis, IGF2BP3 lactylation, lenvatinib resistance, PCK2, serine metabolism

## Abstract

Acquired resistance remains a bottleneck for molecular‐targeted therapy in advanced hepatocellular carcinoma (HCC). Metabolic adaptation and epigenetic remodeling are recognized as hallmarks of cancer that may contribute to acquired resistance. In various lenvatinib‐resistant models, increased glycolysis leads to lactate accumulation and lysine lactylation of IGF2BP3. This lactylation is crucial for capturing PCK2 and NRF2 mRNAs, thereby enhancing their expression. This process reprograms serine metabolism and strengthens the antioxidant defense system. Additionally, altered serine metabolism increases the availability of methylated substrates, such as S‐adenosylmethionine (SAM), for N6‐methyladenosine (m6A) methylation of PCK2 and NRF2 mRNAs. The lactylated IGF2BP3‐PCK2‐SAM‐m6A loop maintains elevated PCK2 and NRF2 levels, enhancing the antioxidant system and promoting lenvatinib resistance in HCC. Treatment with liposomes carrying siRNAs targeting IGF2BP3 or the glycolysis inhibitor 2‐DG restored lenvatinib sensitivity in vivo. These findings highlight the connection between metabolic reprogramming and epigenetic regulation and suggest that targeting metabolic pathways may offer new strategies to overcome lenvatinib resistance in HCC.

## Graphical abstract

This study reveals that in lenvatinib‐resistant hepatocellular carcinoma, increased glycolysis results in lactate accumulation and lysine lactylation of IGF2BP3, which increase the expression of PCK2 and NRF2. This leads to a reprogramming of serine metabolism, S‐adenosylmethionine (SAM) production, RNA m6A modification, and the antioxidant system. The IGF2BP3 lactylation‐PCK2‐SAM‐m6A loop sustains the upregulation of PCK2 and NRF2 expression and ultimately confers lenvatinib resistance.

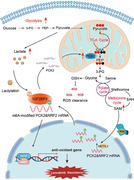

## 1 Introduction

Hepatocellular carcinoma (HCC), a highly aggressive malignancy often diagnosed at advanced stages, has limited treatment options.^[^ ^1^ ^]^ Lenvatinib, a tyrosine kinase receptor inhibitor, represents a frontline therapeutic approach for advanced HCC.^[^ ^2^ ^]^ However, its limited response rate (24.1%)^[^ ^3^ ^]^ and emergent resistance development necessitate molecular mechanism exploration to innovate strategies against drug resistance. Recently, several post‐translational modifications (PTMs), including succinylation,^[^ ^4^ ^]^ asparagine (N)‐linked glycosylation^[^ ^5^ ^]^ and ubiquitination,^[^ ^6^ ^]^ have emerged as pivotal players in the development of resistance to targeted therapies. Histone lysine lactylation (Klac) has been identified as a novel PTM capable of modulating gene transcription or protein function, thereby influencing diverse biological processes, such as tumor formation,^[^ ^7^ ^]^ metabolic reprogramming,^[^ ^8^ ^]^ and resistance to targeted therapies.^[^ ^9^ ^]^ While the abundance and specificity of nonhistone proteins in cellular contexts surpass those of histones,^[^ ^8^, ^10^ ^]^ the extent of lactylated modifications on nonhistone proteins and their subsequent impact on tumor behaviors remain vague.

The reprogramming of cellular metabolism empowers malignant cells to adapt metabolically to intrinsic or extrinsic stress from the microenvironment, resulting in flexibility and high plasticity in macromolecular synthesis and redox homeostasis; this results in acquired therapy resistance.^[^ ^11^ ^]^ Aerobic glycolysis, characterized by elevated glucose uptake and preferential lactate generation, has been implicated in resistance to targeted therapeutic agents.^[^ ^12^ ^]^ A pivotal branch point in glycolysis/gluconeogenesis is the serine synthesis pathway, which contributes to essential biological processes by providing precursors for macromolecule synthesis (including proteins, nucleotides, and lipids).^[^ ^13^ ^]^ Serine serves as a one‐carbon unit donor in the methionine cycle and folate cycle, contributing to nucleotide synthesis and methylation reactions involving histones, 5‐methylcytosine (5‐mC) DNA, and N6‐methyladenosine (m6A) RNA methylation, thereby linking metabolism to epigenetics.^[^ ^13^, ^14^ ^]^ Additionally, activated serine synthesis, coupled with one‐carbon metabolism, generates the main intrinsic antioxidant agents glutathione (GSH) and NADPH to maintain redox balance, conferring chemotherapy resistance.^[^ ^15^ ^]^ RNA m6A modification represents another critical layer of epigenetic regulation in various cellular processes. A previous study suggested that m6A reader IGF2BP3 induces sorafenib resistance in HCC by modulating NRF2 expression.^[^ ^16^ ^]^ However, whether the therapeutic outcomes of targeted drugs in HCC are influenced by glycolysis/gluconeogenesis‐derived serine metabolism and RNA m6A modification remains elusive.

This study reveals that lactate accumulation contributes to IGF2BP3 lactylation (IGF2BP3lac) in lenvatinib‐resistant cells. Lactylation of IGF2BP3 at K76 enhances its binding to m6A‐modified PCK2 and NRF2 mRNAs, increasing their expression. Upregulated PCK2 in lenvatinib‐resistant cells diverts carbon flow from gluconeogenesis to serine metabolism. Our data show that the IGF2BP3lac‐PCK2 axis generates one‐carbon units for SAM biosynthesis, supporting RNA m6A methylation of PCK2 and NRF2 mRNAs and enhancing ROS‐scavenging systems. This IGF2BP3lac‐PCK2‐SAM feedback loop strengthens the antioxidant system and contributes to lenvatinib resistance in HCC. We also highlight the clinical importance of IGF2BP3lac in HCC, as its elevated expression is closely associated with poor outcomes and reduced lenvatinib responsiveness. Our findings suggest that the IGF2BP3lac‐PCK2 axis modulates SAM availability and links metabolic signals to epigenetic modifications, providing potential approaches for reversing lenvatinib resistance in clinical settings.

## 2 Results

### 2.1 Lenvatinib‐Resistant HCC Cell Lines Exhibit Elevated Lactylation Levels

**Figure 1:**
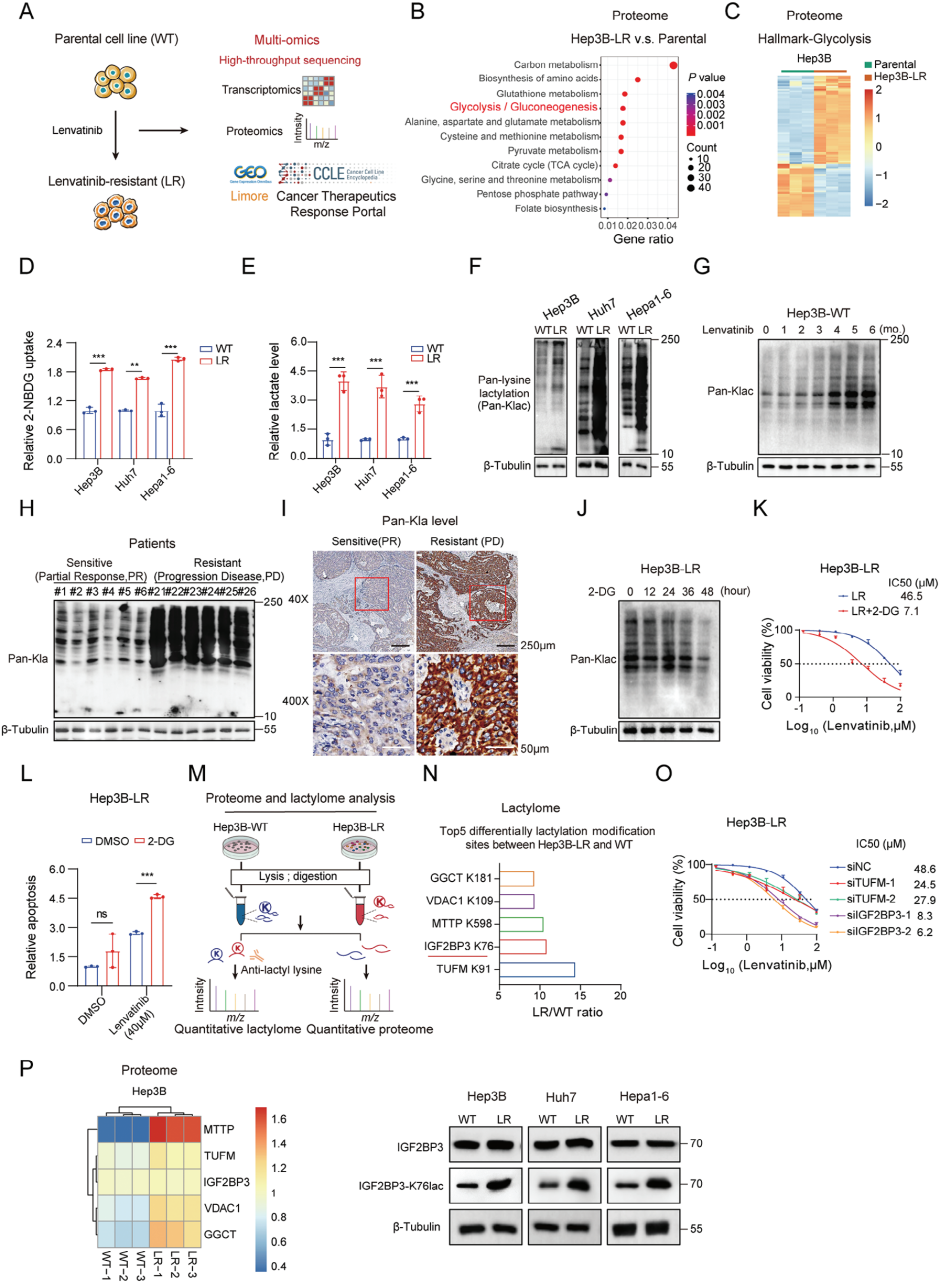
Lenvatinib‐resistant HCC cell lines exhibit elevated lactylation levels. A) Schematic workflow of the multiomics analysis of lenvatinib‐resistant (LR) HCC cell lines. B) Kyoto Encyclopedia of Genes and Genomes (KEGG) analysis highlighted significantly upregulated proteins (log2FC >1) in Hep3B‐LR cells compared with parental cells. C) Heatmap of the expression of proteins related to glycolysis in Hep3B‐LR cells. D) Relative 2‐NBDG uptake (glucose analog; 100 × 10^−6^ m) between lenvatinib‐resistant and parental cell lines. The mean fluorescence intensity of 2‐NBDG uptake was normalized to that of the control. E) Quantification of L‐lactic acid concentrations in parental and lenvatinib‐resistant cells using a lactic acid assay kit. F) Western blot analysis comparing pan‐Klac (pan‐lysine lactylation) levels in parental and lenvatinib‐resistant cells. G) Western blotting of total pan‐Klac levels in Hep3B‐WT cells treated with lenvatinib for various months. H) Western blot analysis of pan‐Klac levels in 12 primary tumor tissues resected after lenvatinib treatment, including six samples from patients with progressive disease (PD) and six from patients with a partial response (PR) to lenvatinib. I) Representative case illustrating high versus low lactylation levels in lenvatinib‐sensitive and lenvatinib‐resistant human HCC tissues assessed by immunohistochemistry (IHC) staining (scale bar: black, 250 µm; white, 50 µm). J) Western blot analysis of pan‐Klac levels in Hep3B‐LR cells treated with 2‐DG (10 × 10^−3^ m) at the indicated times. K) Determination of IC50 values in Hep3B‐LR cells cultured with 2‐DG (10 × 10^−3^ m) for 48 h using the CCK8 assay. L) Analysis of apoptosis in Hep3B‐LR cells treated with lenvatinib (40 × 10^−6^ m) alone or in combination with 2‐DG (10 × 10^−3^ m, glycolysis inhibitor) using flow cytometry with Annexin V staining. M) Flowchart outlining the workflow for proteomics and lactylated proteomics to map differentially expressed proteins and lactylated peptides in Hep3B‐WT and Hep3B‐LR cells. N) Identification of the top five differentially modified sites and proteins between Hep3B‐LR and parental cells using lactylation proteome analysis. O) siRNA screening revealed that IGF2BP3 lactylation may promote lenvatinib resistance in Hep3B‐LR cells. P) Heatmap illustrating the proteomic expression levels of the top five differentially lactylated proteins between Hep3B‐LR cells and parental cells. Western blot analysis of IGF2BP3 expression and the level of IGF2BP3 lactylation in parental and lenvatinib‐resistant cell lines. Statistical significance was determined using two‐tailed unpaired Student's *t*‐test. Each bar in the graph represents the mean ± SD (*n* = 3). Statistical notation: ns (not significant), *p < 0.05, **p < 0.01, ***p < 0.001.

Emerging evidence supports an association between metabolic reprogramming and drug resistance. Tumors often display an enhanced Warburg effect during therapy resistance, leading to rapid nutrient consumption and high lactate secretion.^[^ ^17^ ^]^ To elucidate the molecular mechanisms underlying acquired lenvatinib resistance, we applied a multiomics approach. In addition to the lenvatinib‐resistant Hep3B (Hep3B‐LR) and Huh7 cells (Huh7‐LR) established in our previous study,^[^ ^18^ ^]^ we established another lenvatinib‐resistant Hepa1‐6 cell line (Hepa1‐6‐LR) from the parental Hepa1‐6 cell line. The IC50 for lenvatinib‐resistant cell lines was significantly greater than that for the parental cells (Figure S1A, Supporting Information). The findings were subsequently validated using data from the Cancer Therapeutics Response Portal (CTRP), Liver Cancer Model Repository (LIMORE), and Cancer Cell Line Encyclopedia (CCLE) databases. The experimental procedure is illustrated in the diagram (**Figure** **1**A). Proteomic data revealed a greater level of glycolysis signaling in Hep3B‐LR cells than in parental cells (Figure 1B), demonstrating a marked increase in the protein expression of the glycolysis pathway in Hep3B‐LR cells (Figure 1C). In Hep3B cells treated with lenvatinib, analysis of the Gene Expression Omnibus (GEO) dataset (GSE198845) also revealed marked enrichment of the glycolysis/gluconeogenesis pathway in the resistant group following gene set enrichment analysis (GSEA) (Figure S1B, Supporting Information). GSVA revealed increased glycolytic activity in primary lenvatinib‐resistant cell lines in the LIMORE and CTRP‐CCLE databases. The cells were grouped based on their IC50 values in the databases (Figure S1C,D, Supporting Information), and the names of the cell lines sensitive and resistant to lenvatinib are labeled in Table S7 (Supporting Information). Furthermore, the three lenvatinib‐resistant HCC cell lines showed increased glucose uptake and lactate production (Figure 1D,E). Seahorse analysis also revealed increased glycolytic activity (Figure S1E, Supporting Information), suggesting that increased glycolysis might be a common alteration in resistance to lenvatinib.

Intracellular lactate can drive post‐translational modifications, specifically lysine lactylation (Klac).^[^ ^19^ ^]^ Western blot analysis revealed higher levels of pan‐lysine lactylation (pan‐Klac) in lenvatinib‐resistant cell lines compared to parental cells (Figure 1F). Lactylation levels increased with prolonged lenvatinib administration time. (Figure 1G and Figure S1F, Supporting Information). Evaluation of lactylation levels in 12 HCC cell lines showed that those intrinsically resistant to lenvatinib exhibited higher lactylation levels than sensitive cell lines such as Hep3B, Huh7, and SNU398 (Figure S1G, Supporting Information). Further analysis revealed a significant increase in lactylation levels among patients who experienced disease progression (PD) after lenvatinib treatment, compared to those who achieved a partial response (PR) (Figure 1H,I). Similarly, treatment with 25 × 10^−3^ m sodium L‐lactate (Nala) increased pan‐lactylation in parental cells (Figure S1H, Supporting Information). Conversely, the glycolysis inhibitor 2‐deoxy‐D‐glucose (2‐DG) reduced global lactylation in lenvatinib‐resistant cells, accompanied by a lower IC50 and increased apoptosis (Figure 1J–L and Figure S1I–K, Supporting Information). These findings suggest that lenvatinib resistance may be associated with increased glycolysis and the resulting rise in lactylation. However, the precise impact of lactylation on lenvatinib resistance remains unclear. Using 4D label‐free proteomics, we identified differentially expressed proteins and lactylated peptides proteins in lenvatinib‐resistant cells (Figure 1M). Among the top upregulated lactylated proteins was IGF2BP3, a critical m6A reader implicated in tumor progression and therapeutic resistance (Figure 1N). Subsequent siRNA knockdown screening demonstrated that siIGF2BP3 had a greater impact on lenvatinib sensitivity compared to siTUFM (Figure 1O and Figure S1L, Supporting Information). Therefore, we investigated the role of IGF2BP3 lactylation in lenvatinib resistance. IGF2BP3 showed a substantial increase in lactylation (10.82‐fold) in Hep3B‐LR cells (Figure 1N,P), despite no corresponding increase in protein expression levels, as evidenced by proteomic analysis and Western blot (Figure 1P).

### 2.2 IGF2BP3 Lactylation Confers Lenvatinib Resistance in Vitro and in Vivo

**Figure 2:**
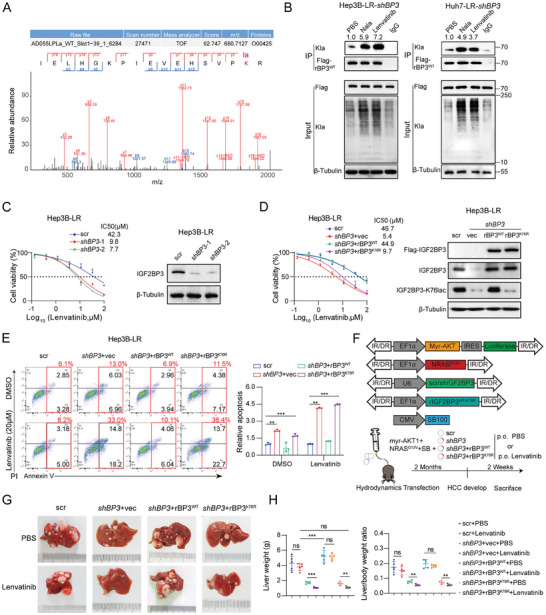
IGF2BP3^K76^ lactylation confers lenvatinib resistance in vitro and in vivo. A) Mapping of IGF2BP3 lactylation sites in Hep3B‐LR cells identified by liquid chromatography‒mass spectrometry (LC‒MS). B) Verification of IGF2BP3 lactylation in Hep3B‐LR‐shBP3 (IGF2BP3) and Huh7‐LR‐shBP3 (IGF2BP3) cells via immunoprecipitation (IP). Cells expressing shRNA‐resistant wild‐type Flag‐rBP3^WT^ (rIGF2BP3^WT^) were treated with PBS, Nala (25 × 10^−3^ m) or lenvatinib (40 × 10^−6^ m) for 24 h, followed by lysis and SDS‐pretreated immunoprecipitation with an anti‐Flag antibody and subsequent Western blot analysis. C) IC50 determination and Western blot analysis of IGF2BP3 levels in Hep3B‐LR cells transduced with shBP3. D) IC50 assay and Western blot analysis of IGF2BP3 lactylation levels in Hep3B‐LR cells transduced with shBP3 plus vec, rBP3^WT^, or the shRNA‐resistant mutation‐rBP3^K76R^ (rIGF2BP3^K76R^) plasmid. E) Apoptosis assessment in Hep3B‐LR cells transduced with shBP3 plus vectors for vec, rBP3^WT^, or rBP3^K76R^. F) Schematic of treatment protocols for a hydrodynamic tail vein‐injected C57BL/6J mouse model established using the scr (scrambled), shBP3, shBP3+rBP3^WT^, or shBP3+rBP3^K76R^ plasmid system. Following tumor nodule formation, the mice were administered PBS or lenvatinib (10 mg k^−1^g) daily via oral gavage for two weeks. AKT, myr‐AKT1; SB, sleeping beauty; NRAS, NRAS^G12V^. G,H) Representative liver images posttherapy (G) and quantification of the tumor weight and liver/body weight ratio (*n* = 5) H). Statistical analysis was conducted using two‐tailed unpaired Student's *t*‐test or one‐way analysis of variance (ANOVA). Each bar represents the mean ± SD (*n* = 3). Statistical notation: ns (not significant), *p < 0.05, **p < 0.01, ***p < 0.001.

We investigated a novel modification pattern involving direct IGF2BP3‐lactylation (IGF2BP3lac) to modulate its function. Liquid chromatography‒mass spectrometry (LC‒MS) identified the lactylation‐modified site of IGF2BP3 at K76 in Hep3B‐LR cells (**Figure** **2**A). To investigate the lactylation of IGF2BP3 in lenvatinib‐resistant cells, we used short hairpin RNA (shRNA) targeting endogenous IGF2BP3 (shIGF2BP3) to eliminate potential interference. Next, we transiently transfected shRNA‐resistant wild‐type IGF2BP3WT (Flag‐rIGF2BP3WT) into Hep3B‐LR‐shIGF2BP3 and Huh7‐LR‐shIGF2BP3 cells and added 25 × 10^−3^ m Nala or 20 × 10^−6^ m lenvatinib to the medium. A subsequent immunoprecipitation (IP) assay confirmed the upregulation of Klac on IGF2BP3 under Nala or lenvatinib treatment (Figure 2B). Next, we explored the biological function of IGF2BP3lac in lenvatinib‐resistant cell lines. IGF2BP3 knockdown (KD) enhanced lenvatinib sensitivity in Hep3B‐LR and Huh7‐LR cells. The efficiency of IGF2BP3 KD using two independent shRNAs in lenvatinib‐resistant cells was verified by western blotting (Figure 2C and Figure S2A, Supporting Information). We then explored whether IGF2BP3lac influences lenvatinib sensitivity by mutating lysine‐76 to arginine (IGF2BP3^K76R^). Hep3B‐LR‐shIGF2BP3 and Huh7‐LR‐shIGF2BP3 cells were transiently transfected with vector, shRNA‐resistant wild‐type (Flag‐rIGF2BP3^WT^), or shRNA‐resistant mutations in Flag‐rIGF2BP3^K76R^(Figure 2D). IGF2BP3 KD potentiated lenvatinib sensitivity, and rIGF2BP3^WT^, but not rIGF2BP3^K76R^, restored lenvatinib resistance (Figure 2D,E and Figure S2B,C, Supporting Information).

The effect of IGF2BP3lac on lenvatinib sensitivity was evaluated in a hydrodynamic transfection mouse model. Hydrodynamic tail vein injection (HTVi) was used to induce HCC tumorigenesis, and protooncogenes (myr‐AKT1 and N‐RAS^G12V^) were coinjected with scr, shIGF2BP3, shIGF2BP3+rIGF2BP3^WT^, or shIGF2BP3+rIGF2BP3^K76R^ plasmids (Figure 2F). After tumor nodule formation, daily lenvatinib administration significantly reduced the tumor burden in the shIGF2BP3 group, as indicated by a marked reduction in liver tumor foci, liver weight, and the liver/body weight ratio (Figure 2G). However, rIGF2BP3^WT^, but not IGF2BP3^K76R^ overexpression, rescued lenvatinib resistance, indicating that IGF2BP3^K76^ lactylation could confer lenvatinib resistance in vivo (Figure 2H).

### 2.3 Increased PCK2 Expression Caused by IGF2BP3^K76^ Lactylation Remodels Redox Homeostasis in Lenvatinib Resistance

**Figure 3:**
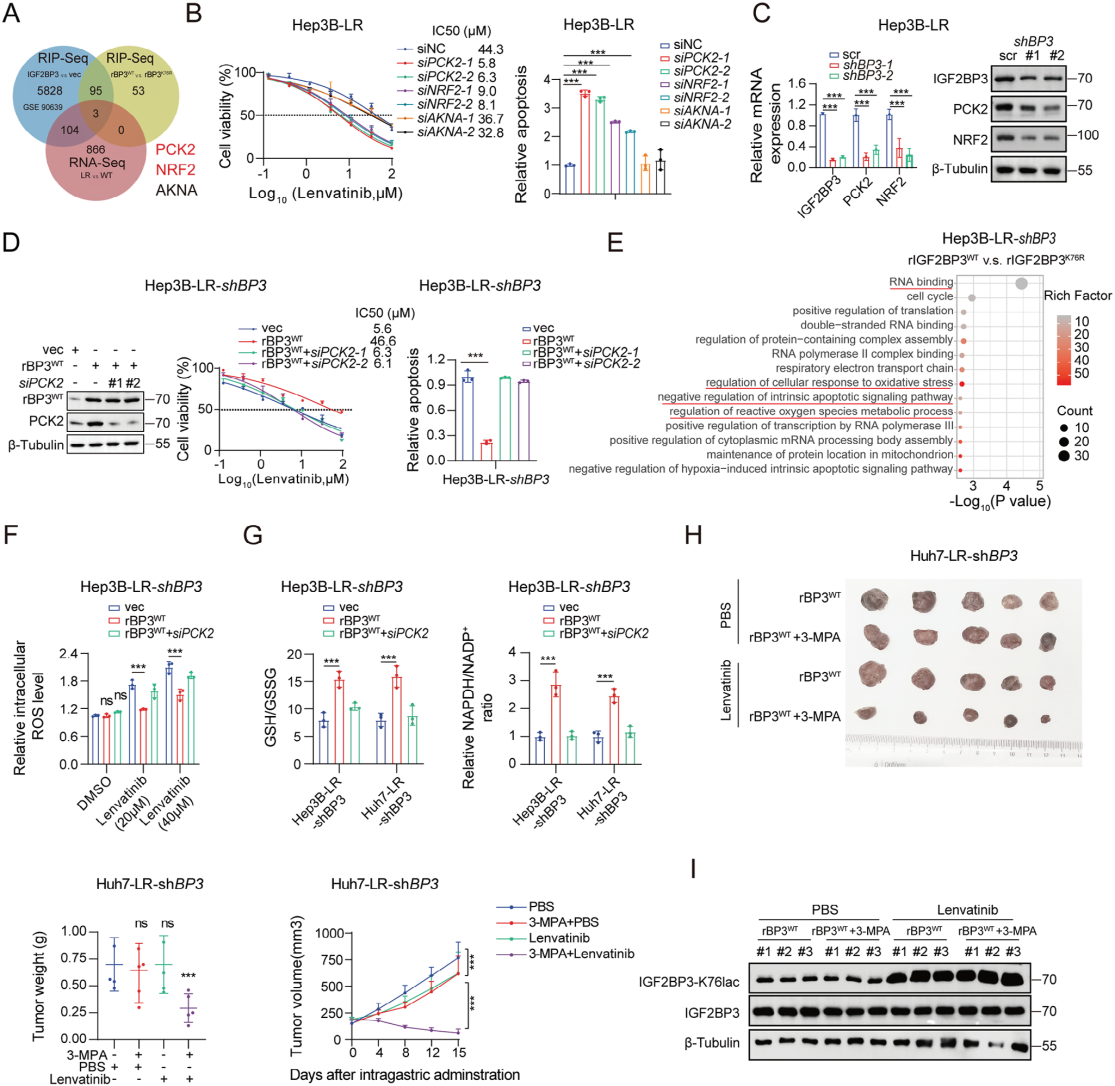
Increased PCK2 expression by IGF2BP3^K76^ lactylation remodels redox homeostasis in lenvatinib resistance. A) Venn diagram illustrating the differentially bound genes in Hep3B‐LR‐shBP3 (IGF2BP3) cells overexpressing rBP3^WT^ (rIGF2BP3^WT^) and rBP3^K76R^ (rIGF2BP3^K76R^). B) SiRNA screening implicates PCK2 and NRF2 as potential targets of lactylated IGF2BP3 in Hep3B‐LR cells. C) Western blot and qPCR analysis of PCK2 and NRF2 expression following IGF2BP3 inhibition in Hep3B‐LR cells. β‐Actin and β‐Tubulin served as loading controls. D) Assessment of the impact of PCK2 inhibition on the IC50 and apoptosis rate (40 × 10^−6^ m; lenvatinib) in Hep3B‐LR‐shBP3 cells transfected with rBP3^WT^. E) KEGG pathway analysis of differential mRNA binding profiles in Hep3B‐LR‐shBP3 cells overexpressing rBP3^WT^ or rBP3^K76R^ plasmids. F,G) Reactive oxygen species (ROS) levels F) and the GSH/GSSG and NADPH/NADP^+^ ratios G) following PCK2 inhibition in Hep3B/Huh7‐LR‐shBP3 cells overexpressing rBP3^WT^. (H) Subcutaneous tumor growth (Huh7‐LR‐sh‐BP3 cells overexpressing rBP3^WT^) in BALB/c nude mice (*n* = 5 per group). Tumors were treated via intragastric administration of vehicle (PBS), 3‐MPA (3‐mercaptopicolinic acid) (30 mg k^−1^g), or combination with lenvatinib (10 mg k^−1^g) daily. The tumor volumes and weights were monitored bi‐daily and are presented as the means ± SD. I) Western blot analysis of IGF2BP3 lactylation levels in the Huh7‐LR cell‐derived orthotopic HCC model treated with 3‐MPA alone or in combination with lenvatinib. Statistical analysis was conducted using one‐way analysis of variance (ANOVA). Each bar represents the mean ± SD. Statistical notations: ns, not significant, *p < 0.05, **p < 0.01, ***p < 0.001.

We investigated the underlying mechanisms of IGF2BP3 lactylation (IGF2BP3lac) in lenvatinib resistance. Using RNA immunoprecipitation sequencing (RIP‐seq) of Hep3B‐LR‐shIGF2BP3 cells expressing rIGF2BP3^WT^ or rIGF2BP3^K76R^, we identified three RNAs (PCK2, NRF2, and AKNA) that preferentially interact with lactylated IGF2BP3. These RNAs were consistently observed across various datasets, including RNA‐seq of Hep3B‐LR versus WT cells, and RIP‐seq of Flag‐IGF2BP3 in 293T cells (GSE90639) (**Figure** **3**A). siRNA‐mediated knockdown of PCK2 or NRF2 significantly increased lenvatinib sensitivity in lenvatinib‐resistant cells (Figure 3B and Figure S3A–C, Supporting Information), underscoring the role of these genes in resistance mechanisms. IGF2BP3 KD markedly reduced PCK2 and NRF2 expression at both the mRNA and protein levels (Figure 3C and Figure S3D, Supporting Information), indicating their regulatory interdependence. Given our previous findings highlighting the role of NRF2 in resistance mechanisms,^[^ ^18^ ^]^ we focused on the involvement of PCK2 in gluconeogenesis^[^ ^20^ ^]^ and its potential to mitigate oxidative stress, which differs from the direct regulation of stress responses by NRF2 in lenvatinib‐resistant cell lines. To assess the role of PCK2 in IGF2BP3lac‐mediated lenvatinib resistance, we compared lenvatinib sensitivity in cells overexpressing rIGF2BP3^WT^ with and without siRNA‐mediated PCK2 knockdown. rIGF2BP3^WT^ overexpression partially restored the decrease in PCK2 levels induced by IGF2BP3 KD, increasing IC50 values and decreasing lenvatinib‐induced apoptosis in Hep3B‐LR‐shIGF2BP3 and Huh7‐LR‐shIGF2BP3 cells. Conversely, PCK2 inhibition markedly reduced the capacity of IGF2BP3^WT^ to confer lenvatinib resistance (Figure 3D and Figure S3E,F, Supporting Information).

PCK2 serves as a key enzyme in gluconeogenesis, providing substrates for serine synthesis,^[^ ^21^ ^]^ which modulates redox homeostasis.^[^ ^13^, ^20^ ^]^ Our KEGG enrichment analysis of rIGF2BP3^WT^ and rIGF2BP3^K76R^ also revealed that IGF2BP3 facilitates increased antioxidant stress capacity (Figure 3E). To confirm the hypothesized role of IGF2BP3lac in improving antioxidant capacity, we measured the cellular levels of GSH and NADPH and observed a significant increase in these antioxidants in lenvatinib‐resistant cells (Figure S3G, Supporting Information). Given the role of PEPCK (PCK1 or PCK2) in regulating antioxidants in tumor cells, we investigated whether IGF2BP3lac influences redox homeostasis by modulating PCK2 expression. Knockdown of PCK2 significantly reduced glutathione GSH/NADPH levels in lenvatinib‐resistant cells, coinciding with increased reactive oxygen species (ROS) (Figure S3H–J, Supporting Information). Moreover, PCK2 knockdown (KD) abrogated the increase in GSH/NADPH induced by rIGF2BP3^WT^ expression in IGF2BP3‐KD cells, concomitantly increasing ROS levels (Figure 3F,G and Figure S3K, Supporting Information). In vivo administration of 3‐MPA, a PCK2 inhibitor, to subcutaneous xenograft models with rIGF2BP3^WT^‐overexpressing Huh7‐LR‐shIGF2BP3 cells markedly increased lenvatinib sensitivity. In a subcutaneous murine tumor model, lenvatinib provoked significant IGF2BP3 lactylation. However, upon PCK2 inhibition, IGF2BP3 could not facilitate lenvatinib resistance (Figure 3H–I). These findings underscore the pivotal role of PCK2 in IGF2BP3lac‐mediated lenvatinib resistance in HCC.

### 2.4 IGF2BP3 Modulates PCK2 and NRF2 Expression in an m6A‐Dependent Manner

We further investigated the mechanisms that regulate PCK2 and NRF2 expression in lenvatinib‐resistant cells. RIP‐qPCR analysis confirmed the increased binding affinity of IGF2BP3 for PCK2 and NRF2 mRNAs in lenvatinib‐resistant cells (Figure S4A,B, Supporting Information). To clarify the underlying molecular interactions, we analyzed the RIP‐seq data and predicted the m6A motifs of the PCK2 and NRF2 transcripts using the SRAMP tool (http://www.cuilab.cn/sramp), identified four potential m6A modification sites in both the PCK2 (P1, P2, P3, P4) and NRF2 (N1, N2, N3, N4) transcripts (Figure S4C,D, Supporting Information), and performed MeRIP‐qPCR for further corroboration (Figure S4E, Supporting Information). m6A modification of PCK2 occurred mainly and was recognized by IGF2BP3 at sites P3 and P4, whereas that of NRF2 occurred mainly at sites N1 and N2 (Figure S4E, Supporting Information). Two m6A modification sites for PCK2 (P3 and P4) and NRF2 (N1 and N2) were validated via a step‐by‐step mutation of the reporter plasmid (Figure S4F, Supporting Information). Subsequently, in vitro RNA pull‐down assays revealed that m6A‐modified PCK2 and NRF2 RNA probes bind to IGF2BP3 and that this interaction is disrupted by A‐to‐T point mutations at these m6A sites (Figure S4G, Supporting Information). We also evaluated the role of m6A modification in regulating PCK2 and NRF2 mRNA expression. We compared the expression of m6A writer, eraser, and reader proteins between the parental and lenvatinib‐resistant cell lines. Notably, the m6A methyltransferases METTL3, METTL14, and WTAP presented increased RNA levels in resistant cells (Figure S4H, Supporting Information). However, only interference with METTL3 resulted in a decrease in PCK2 and NRF2 expression, as opposed to interference with METTL14 or WTAP (Figure S4I, Supporting Information). RIP‐qPCR using an IGF2BP3‐specific antibody revealed that the binding of IGF2BP3 to PCK2 and NRF2 mRNAs was significantly suppressed by METTL3 KD in resistant cells (Figure S5A, Supporting Information). This effect was accompanied by a marked reduction in m6A abundance in these mRNAs (Figure S5B,C, Supporting Information). The actinomycin D assay further confirmed that mRNA stability was attenuated in cells with IGF2BP3 or METTL3 KD (Figure S5D–G, Supporting Information). Finally, polysome fractionation experiments indicated that IGF2BP3 KD resulted in a decrease in the polysome fractions of PCK2 and NRF2 mRNAs (Figure S5H, Supporting Information), suggesting a role for IGF2BP3 in regulating mRNA stability and translation. Collectively, these findings elucidated the specific m6A sites in PCK2 and NRF2 mRNA transcripts and demonstrated that IGF2BP3 regulates these transcripts in an m6A‐dependent manner.

### 2.5 Lactylation of IGF2BP3^K76^ Enhances the Capture of m6A‐Modified RNA in Lenvatinib Resistance

**Figure 4:**
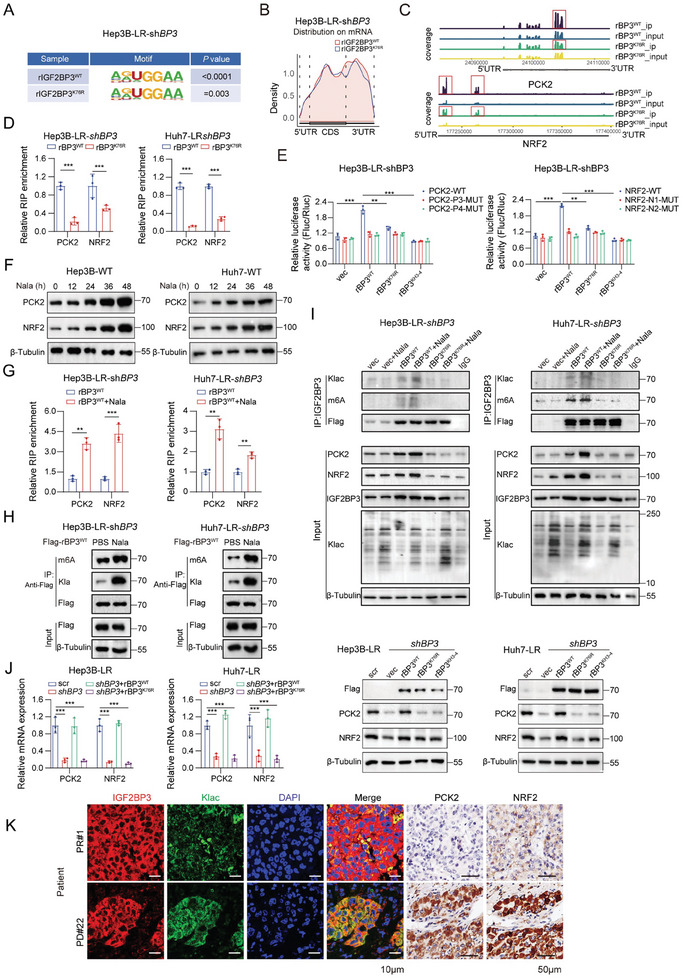
Lactylation of IGF2BP3^K76^ enhanced the capture of m6A‐modified RNA in lenvatinib resistance.A) Sequence analysis identifying the highly enriched motif within m6A peaks from RIP‐seq data in Hep3B‐LR‐shBP3 (shIGF2BP3) cells overexpressing rBP3^WT^ (rIGF2BP3^WT^) or rBP3^K76R^ (rIGF2BP3^K76R^) plasmids. B) Density distribution analysis of binding targets to rBP3^WT^ or rBP3^K76R^. C) Gene plots representing PCK2 and NRF2 binding abundance in Hep3B‐LR‐shBP3 cells under conditions of rBP3^WT^ and rBP3^K76R^ overexpression. D) RIP assays using an IGF2BP3 antibody, followed by qPCR in Hep3B/Huh7‐LR‐shBP3 cells with ectopically rBP3^WT^ or rBP3^K76R^. E) Relative luciferase activity of PCK2 (P3 or P4 m6A modification sites mut) or NRF2 (N1 or N2 m6A modification sites mut) reporters in Hep3B‐LR‐shBP3 cells with forced expression of Flag‐rBP3^WT^, Flag‐rBP3^K76R^, or Flag‐rBP3^KH3‐4 mut^ plasmid. The Fluc/Rluc ratio (representing luciferase activity) of PCK2‐WT or NRF2‐WT with empty vector was used for normalization. F) Western blot analysis of indicated proteins from whole‐cell lysates of Hep3B/Huh7 parental cells treated with Nala (25 × 10^−3^ m) for 24 h. G) Flag‐rBP3^WT^ transfection into Hep3B/Huh7‐LR‐shBP3 cells for 24 h, followed by Nala (25 × 10^−3^ m) treatment and subsequent RIP assays with anti‐Flag antibody and qPCR. H) Flag‐rBP3^WT^ transfected into Hep3B/Huh7‐LR‐shBP3 cells for 24 h, followed by Nala (25 × 10^−3^ m) treatment for 24 h. Post 254 nm UV‐crosslinking, cell lysates underwent SDS pre‐treated immunoprecipitation with anti‐Flag antibody, and western blotting for target detection. I) Flag‐rBP3^WT^ and Flag‐rBP3^K76R^ transfected into Hep3B/Huh7‐LR‐shBP3 cells for 24 h, then treated with Nala (25 × 10^−3^ m) for 24 h. Post 254 nm UV‐crosslinking, lysates were used for SDS pre‐treated immunoprecipitation with anti‐Flag antibody, followed by Western blot for target identification. J) Protein and mRNA levels of PCK2, NRF2 in Hep3B/Huh7‐LR cells transfected with shBP3, and rescued with Flag‐rBP3^WT^, Flag‐rBP3^K76R^, or Flag‐rBP3^KH3‐4 mut^. K) Confocal microscopy examination of IGF2BP3 (red) and pan‐Klac (green) co‐localization (scale bar, white,10 µm), with DAPI (blue) indicating the nucleus. Co‐localization analysis utilized ImageJ. Representative cases exhibit varying levels of PCK2 and NRF2 in human lenvatinib‐sensitive and ‐resistant HCC tissues, analyzed via immunohistochemistry (IHC) staining (scale bar: black, 50 µm). Statistical analyses were performed using two‐tailed unpaired Student's *t*‐test or one‐way analysis of variance (ANOVA). Each bar in the graph represents the mean ± SD. *p < 0.05, **p < 0.01, ***p < 0.001.

Metabolites enable specific post‐translational modifications (PTMs) of proteins that can dramatically alter protein function.^[^ ^22^ ^]^ Several types of post‐translational modifications with important functions have been identified on m6A writers, such as SUMOylation of METTL3 and lactylation of METTL16 or METTL3, which have been reported to influence methyltransferase activity.^[^ ^10^, ^23^ ^]^ Next, we investigated whether direct lactylation of IGF2BP3 is involved in modulating its function as an m6A reader. RIP‐seq comparing wild‐type IGF2BP3 (IGF2BP3^WT^) and its K76R mutant revealed predominant binding in coding sequences (CDSs) and 3′‐untranslated regions, with diminished binding in the K76R mutant (**Figure** **4**A,B). Functional enrichment analysis of differentially bound RNA between the IGF2BP3^WT^ and IGF2BP3^K76R^ groups revealed a focus on RNA binding and translation regulation (Figure 3E). These findings suggest that lactylation is essential for the RNA‐binding function of IGF2BP3. RIP‐seq data revealed reduced m6A peak enrichment in PCK2 and NRF2 mRNAs in IGF2BP3^K76R^ cells (Figure 4C), indicating the dynamic regulation of these mRNAs by IGF2BP3lac. RIP‒qPCR analysis identified NRF2 and PCK2 as significant targets of IGF2BP3lac (Figure 4D). Moreover, IGF2BP3^WT^‐promoted luciferase expression of the PCK2‐WT or NRF2‐WT reporter was strongly impaired by the K76R mutation and completely diminished by mutations of GXXG to GEEG in the KH3‐4 domains (Figure 4E).^[^ ^24^ ^]^ As expected, ectopic IGF2BP3^WT^ induced a significant increase in Fluc activity in the wild‐type reporter (PCK2 and NRF2); however, this increase was largely impaired by mutations in the m6A sites of PCK2 (P3 or P4 RNA oligos) or NRF2 (N1 or N2 RNA oligos) (Figure 4E). Furthermore, elevated lactate levels in lenvatinib‐resistant cells and increased IGF2BP3lac levels prompted us to investigate the role of lactate in PCK2 and NRF2 upregulation. Addition of 25 × 10^−3^ m Nala to Hep3B‐WT and Huh7‐WT cell culture media significantly increased PCK2 and NRF2 expression (Figure 4F). Subsequent RIP‐qPCR using anti‐Flag‐IGF2BP3 confirmed the increased binding of IGF2BP3 to PCK2 and NRF2 mRNAs with Nala supplementation (Figure 4G). Therefore, we deduced that lactic acid in lenvatinib‐resistant cells was at least partially responsible for PCK2 and NRF2 upregulation.

To further determine whether lactylation affects the binding of IGF2BP3 to m6A‐modified RNA, we transfected vectors with Flag‐rIGF2BP3^WT^, Flag‐rIGF2BP3^K76R^, or Vec into IGF2BP3‐KD cells supplemented with 25 ×10^−3^ m Nala. Immunoprecipitation with an anti‐Flag antibody, followed by Western blotting using anti‐Klac and anti‐m6A antibodies, revealed that m6A‐modified RNAs bound more abundantly to Flag‐rIGF2BP3^WT^‐Nala than to Flag‐rIGF2BP3^WT^ (Figure 4H). Interestingly, the overexpression of rIGF2BP3^WT^ with Nala, but not the lactylation‐deficient IGF2BP3^K76R^ mutant, resulted in the highest lactylation levels and significantly increased m6A‐modified RNA binding (Figure 4I). In lenvatinib‐resistant cells, IGF2BP3 knockdown (KD) significantly reduced PCK2 and NRF2 expression, whereas their decreased expression was rescued by rIGF2BP3^WT^ but not by rIGF2BP3^K67R^ or mutations of GXXG to GEEG in the KH3‐4 domains (Figure 4J). Additionally, increased IGF2BP3lac expression, along with increased PCK2 and NRF2 expression, was confirmed in lenvatinib‐resistant clinical specimens (Figure 4K).

### 2.6 Lactylated IGF2BP3 Drives Serine Metabolism Reprogramming via PCK2 Upregulation in Lenvatinib‐Resistant HCC

**Figure 5:**
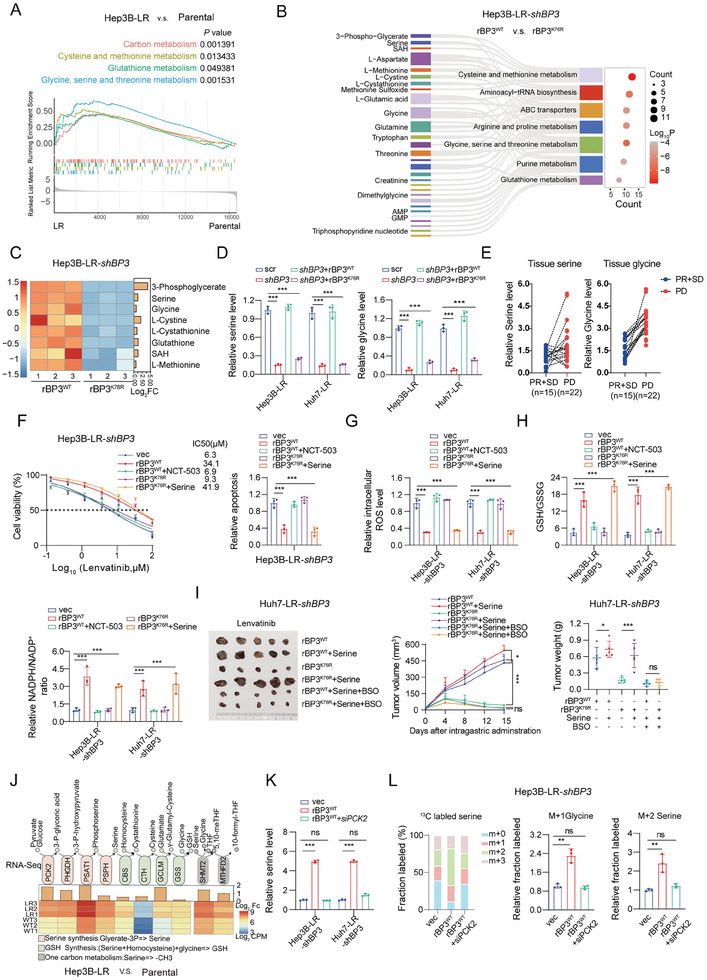
Lactylated IGF2BP3 drives serine metabolism reprogramming via PCK2 upregulation in lenvatinib‐resistant HCC. A) GSEA of RNA‐seq data from Hep3B‐LR cells versus parental cells, highlighting the glycine, serine, and threonine metabolism pathways, along with glutathione metabolism. B) Metabolite analysis via LC‒MS in Hep3B‐LR‐shBP3 (IGF2BP3) cells expressing rBP3^WT^ (rIGF2BP3^WT^) (*n* = 3) or rBP3^K76R^ (rIGF2BP3^K76R^) (*n* = 3). Pathway enrichment analysis was performed using MetaboAnalyst 5.0 with KEGG and the Small Molecule Pathway Database. The circle size represents the number of enriched metabolites, and the line thickness indicates the number of common metabolites between groups. C) Heatmap displaying variations in intermediate metabolites of serine metabolism in Hep3B‐LR‐shBP3 cells with rBP3^WT^ and rBP3^K76R^ expression (*n* = 3 biologically independent samples). D) Total intracellular serine and glycine levels measured by LC‒MS in Hep3B‐LR and Huh7‐LR cells transduced with shBP3 plus vectors for vec, rBP3^WT^, or rBP3^K76R^ overexpression and cultivated in complete medium. E) Serine and glycine levels analyzed by LC‒MS in HCC patient samples (PR+SD = 15, PD = 22). F–H) Analysis of the IC50 values and apoptosis rates F), ROS levels G), GSH/GSSG ratios and NADPH/NADP+ ratios H) in Hep3B/Huh7‐LR‐shBP3 cells transfected with vec, rBP3^WT^, rBP3^WT^ + NCT‐503 (20 × 10^−6^ m), rBP3^K76R^, or rBP3^K76R^ + serine (400 × 10^−6^ m). I) Subcutaneous tumor growth in BALB/c nude mice (n = 5 per group) injected with Huh7‐LR‐shBP3+rBP3^WT^ or Huh7‐LR‐shBP3+rBP3^K76R^ cells and treated with PBS, serine (20 mg k^−1^g), or buthionine sulfoximine (BSO, an ROS scavenging inhibitor) (450 mg k^−1^g) in conjunction with daily lenvatinib (10 mg k^−1^g). The tumor weights and volumes are presented for each group.J) Heatmap illustrating the expression of metabolic genes related to serine metabolism identified via RNA‐seq analysis in parental and Hep3B‐LR cells. K) Total intracellular serine levels assessed by LC‒MS after PCK2 inhibition in Hep3B/Huh7‐LR‐shBP3 cells transfected with rBP3^WT^. L) Analysis of ^13^C‐labeled metabolites by liquid chromatography‒mass spectrometry (LC‒MS) after Hep3B‐LR‐shBP3 cells with vec, rBP3^WT^ or rBP3^WT^+siPCK2 were incubated with ^13^C‐glucose for 12 h. Left: abundance of serine and glycine after glucose tracing. Right: quantified fraction of labeled m+2 serine and m+1 glycine (*n* = 3 biologically independent samples). Statistical analysis was performed using one‐way analysis of variance (ANOVA). Each bar in the graph represents the mean ± SD (*n* = 3). Statistical notation: ns (not significant), *p < 0.05, **p < 0.01, ***p < 0.001.

We sought to determine the biological implications of IGF2BP3lac in modulating lenvatinib resistance. Various metabolites pivotal for activating antioxidant programs, such as GSH, NADPH, and serine, are implicated in drug resistance.^[^ ^19^ ^]^ GSEA of Hep3B‐LR (**Figure** **5**A) and Huh7‐LR (Figure S6A, Supporting Information) versus parental transcriptome data, along with data from sorafenib‐resistant patient samples (Figure S1B, Supporting Information), revealed pronounced enrichment in glycine, serine, and threonine metabolism in the drug‐resistant cohort. Given that PCK2 supplies carbon for gluconeogenesis and several anabolic pathways (glyceroneogenesis, PPP, and serine synthesis), we investigated whether IGF2BP3lac affects serine metabolism in lenvatinib‐resistant cell lines through LC‒MS analysis of Hep3B‐LR‐shIGF2BP3 cells with forced expression of rIGF2BP3^WT^ or rIGF2BP3^K76R^.

Targeted LC‒MS‐based metabolomic analysis revealed global metabolic alterations between IGF2BP3^WT^ and IGF2BP3^K76R^, with IGF2BP3^WT^ showing elevated glycine, serine, and threonine metabolism (Figure 5B and Figure S6B, Supporting Information). Among the differentially abundant metabolites in the corresponding pathway, the level of serine, an oncogenesis‐supportive and chemoresistance metabolite,^[^ ^25^ ^]^ was significantly increased in the IGF2BP3^WT^ group relative to the IGF2BP3^K76R^ group (Figure 5C). To validate whether IGF2BP3lac drives serine synthesis, we assessed serine and glycine levels after IGF2BP3 inhibition. LC‒MS analysis revealed that IGF2BP3 KD reduced serine and glycine levels, which was reversed by rIGF2BP3^WT^ but not rIGF2BP3^K76R^ (Figure 5D). Elevated serine and glycine levels in lenvatinib‐resistant cells and patients further support these findings (Figure 5E and Figure S6C, Supporting Information).

Considering the association between serine synthesis and chemotherapy resistance, we investigated the effects of serine restriction on lenvatinib sensitivity. Serine deprivation increased lenvatinib sensitivity and induced apoptosis in resistant cells (Figure S6D–E, Supporting Information). Serine metabolism supports cancer progression by maintaining redox balance and providing substrates for macromolecule synthesis and methylation. Our previous work highlighted enhanced ROS scavenging in lenvatinib‐resistant cells.^[^ ^18^ ^]^ Serine deprivation notably reduced the GSH/GSSG and NADPH/NADP+ ratios while increasing ROS accumulation (Figure S6F,G, Supporting Information). Importantly, qPCR analysis revealed that key enzymes involved in serine metabolism (PCK2, PHGDH) and classic antioxidative stress‐related pathways (NRF2, HMOX1, GPX4, NQO1) were significantly upregulated in lenvatinib‐resistant cells (Figure S6H, Supporting Information). Importantly, IGF2BP3 KD resulted in elevated ROS levels (Figure S6I, Supporting Information) and reduced antioxidant levels, which were restored by rIGF2BP3^WT^ but not rIGF2BP3^K76R^ (Figure S6J,K, Supporting Information). Treatment with buthionine sulfoximine (BSO) in rIGF2BP3^WT^ cells and N‐acetyl‐L‐cysteine (NAC) in rIGF2BP3^K76R^ cells further confirmed the antioxidant‐dependent nature of IGF2BP3lac‐mediated lenvatinib resistance (Figure S6L, Supporting Information).

We investigated whether IGF2BP3lac regulated serine biosynthesis, thereby enhancing the antioxidant capacity of drug‐resistant cells and promoting lenvatinib resistance. Treatment of IGF2BP3^WT^ cells with a PHGDH inhibitor (NCT‐503) blocked serine synthesis and reversed improvements in redox homeostasis, as evidenced by increased ROS and decreased GSH/GSSG and NADPH/NADP+ ratios (Figure 5F–H and Figure S7A–C, Supporting Information). In contrast, IGF2BP3^K76R^‐mediated restraint of serine biosynthesis markedly decreased the production of GSH/GSSG and NADPH/NADP+ but was completely restored by serine addition, accompanied by decreased ROS levels (Figure 5F–H and Figure S7A–C, Supporting Information). Additionally, following intervention with IGF2BP3, the mRNA expression of genes involved in serine metabolism, GSH metabolism and antioxidant enzymes significantly changed (Figure S7D, Supporting Information). In vivo, serine supplementation enhanced the antioxidant capacity of IGF2BP3^K76R^ xenografts. Moreover, BSO treatment negated the effect of IGF2BP3lac on lenvatinib sensitivity in Huh7‐LR‐shIGF2BP3‐derived xenografts (Figure 5I and Figure S6M, Supporting Information). These findings collectively confirmed that the upregulation of IGF2BP3lac ameliorated antioxidant capacity by modulating serine metabolism pathways, which are critical for increasing lenvatinib resistance.

We further explored the mediators through which IGF2BP3lac fuels serine synthesis in lenvatinib‐resistant cells. Further analysis of the transcriptional data revealed PCK2 and PHGDH as the dominant metabolic genes involved in serine synthesis, with GCLM and SHMT2 as key downstream genes (Figure 5J). Surprisingly, the IC50 values of lenvatinib were decreased by 80–90% (PCK2 KD) and 70–80% (PHGDH KD) in Huh7‐LR and Hep3B‐LR cells compared with those of the scrambled controls (Figure S7E, Supporting Information). Compared with PHGDH, PCK2 inhibition effectively mitigated the elevated serine and glycine levels and GSH/NADPH ratios in lenvatinib‐resistant cells (Figure S7F,G, Supporting Information). GSEA of the transcriptional data of Hep3B‐LR cells and PCK2‐KD cells confirmed the role of PCK2 in serine synthesis (Figure S7H, Supporting Information). Given the reported role of PEPCK in the upregulation of PHGDH protein expression, we focused on PCK2. Subsequently, targeted LC‒MS revealed that PCK2 KD dramatically decreased the intracellular serine level in the IGF2BP3^WT^ (Figure 5K) and lenvatinib‐resistant cells (Figure S7I, Supporting Information). Using LC‒MS‐based 13C‐glucose stable isotope tracing, we found that rIGF2BP3^WT^ increased the M+2 serine and M+1 glycine levels, but this effect was reversed by PCK2 inhibition (Figure 5L). These findings underscore the role of IGF2BP3lac‐PCK2 in mediating serine synthesis and redox homeostasis during lenvatinib resistance.

### 2.7 IGF2BP3lac‐PCK2 Supports S‐Adenosylmethionine (SAM) Biosynthesis and RNA m6A Modification to Contribute to Lenvatinib Resistance

**Figure 6:**
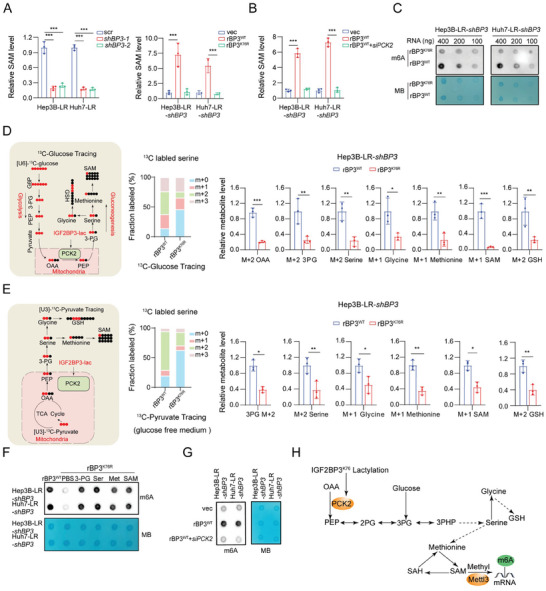
Lactylation of IGF2BP3‐PCK2 supports SAM biosynthesis, and RNA m6A modification contributes to lenvatinib resistance. A) Assessment of S‐adenosylmethionine (SAM) levels by ELISA in Hep3B‐LR and Huh7‐LR cells. The cells were transduced with shBP3 (IGF2BP3) or plus vectors for vec, rBP3^WT^ (rIGF2BP3^WT^), or rBP3^K76R^ (rIGF2BP3^K76R^). B) Evaluation of SAM levels following PCK2 inhibition in Hep3B/Huh7‐LR‐shBP3 cells transfected with rBP3^WT^. C) RNA m6A dot blot assays in Hep 3B/Huh7‐LR‐shBP3 cells transfected with rBP3^WT^ or rBP3^K76R^. Methylene blue (MB) was used as a loading control. D) Analysis of ^13^C‐labeled metabolites by liquid chromatography‒mass spectrometry (LC‒MS) after Hep3B‐LR‐shBP3 cells overexpressing rBP3^WT^ or rBP3^K76R^ were incubated with ^13^C‐glucose for 12 h. The schematic diagram illustrates the conversion of U‐[^13^C]‐glucose into various metabolites, with LC‒MS profiles of M+2 OAA, M+2 3PG, M+2 serine, M+1 glycine, M+1 methionine, M+1 SAM and M+2 GSH (n = 3 biologically independent samples). E) Similar analysis as in D) but with cells incubated with ^13^C‐pyruvate for 12 h (without glucose), showing the metabolic conversion and LC‒MS profiles of M+2 3PG, M+2 serine, M+1 glycine, M+1 methionine, M+1 SAM and M+2 GSH (*n* = 3 biologically independent samples). F) RNA m6A dot blot assays were conducted in Hep3B/Huh7‐LR‐shBP3 cells overexpressing rBP3^WT^ or rBP3^K76R^. Cells transfected with rBP3^K76R^ were treated with 3PG (0.75 × 10^−3^ m), serine (400 × 10^−6^ m), methionine (100 × 10^−6^ m), or SAM (50 × 10^−6^ m) for 24 h. G) Global m6A levels were assessed through m6A dot blot assays following PCK2 inhibition in Hep3B/Huh7‐LR‐shBP3 cells transfected with rBP3^WT^. H) Schematic overview depicting the mechanism whereby IGF2BP3 lactylation‐induced m6A modification is dependent on SAM accumulation derived from the serine synthesis pathway. Statistical analysis was conducted using two‐tailed unpaired Student's *t*‐test or one‐way analysis of variance (ANOVA). Each bar in the graph represents the mean ± SD (*n* = 3). Statistical notations: *p < 0.05, **p < 0.01, ***p < 0.001.

Increased serine synthesis in lenvatinib‐resistant cells potentially augments the one‐carbon metabolism pathway, thereby supporting the production of SAM, a key methyl donor for global methylation.^[^ ^13^, ^26^ ^]^ This hypothesis was supported by the elevated SAM levels in lenvatinib‐resistant cells, as verified using an ELISA kit (Figure S8A, Supporting Information). KEGG pathway analysis of our RNA‐seq data revealed that serine metabolism, RNA methylation, and SAM metabolic processes were significantly enriched in PCK2‐KD cells (Figure S8B, Supporting Information). We evaluated SAM levels following IGF2BP3 or PCK2 inhibition. The reduction in SAM caused by IGF2BP3 KD was almost completely reversed by IGF2BP3^WT^ but not by IGF2BP3^K76R^ (**Figure** **6**A). Specifically, PCK2 inhibition significantly decreased intracellular SAM levels, and the expression of IGF2BP3^WT^ failed to rescue SAM levels after PCK2 inhibition (Figure 6B and Figure S8C, Supporting Information), suggesting that IGF2BP3lac drives serine synthesis and that subsequent SAM upregulation is contingent on PCK2 expression.

SAM is a universal methyl donor for methylation reactions, including 5‐methylcytosine (5‐mC) in DNA, N6‐methyladenosine (m6A) in RNA, and histone methylation.^[^ ^27^ ^]^ Dot blot analysis revealed increased global m6A levels in lenvatinib‐resistant cells, with minor increases in 5‐mC and histone methylation (Figure S8D,E, Supporting Information). Immunofluorescence staining also revealed increased m6A levels in lenvatinib‐resistant patients (Figure S8F, Supporting Information), underscoring the potential role of m6A in lenvatinib resistance. In addition, IGF2BP3lac did not significantly affect DNA or histone methylation (Figure S8G, Supporting Information), whereas IGF2BP3^K76R^ reduced RNA m6A methylation (Figure 6C). The sensitivity of lenvatinib‐resistant cells was restored by the RNA m6A methylation inhibitor 3‐deazaadenosine (DAA), whereas histone and DNA methylation inhibitors had modest effects on the IC50 (Figure S8H, Supporting Information). Therefore, in this study, we focused on N6‐methyladenosine (m6A) RNA methylation.

Serine synthesis originates from the 3‐phosphoglycerate (3‐PG) transition, which is derived from gluconeogenesis and glycolysis. Given the enhanced glycolytic activity in lenvatinib‐resistant cells (Figure 1B,C) and the role of PCK2 in promoting glycolytic/gluconeogenic intermediates,^[^ ^28^ ^]^ we investigated the impact of IGF2BP3lac on glycolysis and serine synthesis. IGF2BP3lac did not increase glucose uptake or lactate production (Figure S9A,B, Supporting Information). PCK2 inhibition also did not affect these parameters (Figure S9C,D, Supporting Information). Using LC‒MS‐based ^13^C‐glucose stable isotope tracing, we found that IGF2BP3lac increased M+2 3PG, M+2 serine, M+1 methionine, and M+1 SAM levels, diverting glucose to serine synthesis (Figure 6D). However, ^13^C‐glucose tracing did not reveal an increase in M+3 serine levels, suggesting that glycolysis does not contribute to increased serine levels in lenvatinib‐resistant cells (Figure S9E, Supporting Information). U‐[13C]‐pyruvate tracing confirmed the significant role of IGF2BP3lac in increasing the incorporation of labeled ^13^C into M+2 PGs, M+2 serine, M+1 methionine, and M+1 SAM (Figure 6E), as well as in promoting glutathione (GSH) generation under lenvatinib treatment (Figure 6D,E).

To validate the reliance of m6A modification on IGF2BP3lac, we supplemented IGF2BP3^K76R^ cells with SAM and related metabolites, including PEP, 3PG, serine, and methionine. Exogenous serine and SAM significantly increased m6A levels (Figure 6F). IGF2BP3lac increased m6A modifications, whereas PCK2 inhibition negated this effect (Figure 6G,H, and Figure S8I, Supporting Information). These results underscore the role of lactylated IGF2BP3 in directing gluconeogenic intermediates toward serine synthesis, thereby facilitating SAM generation, inducing RNA m6A modifications, and promoting a lenvatinib‐resistant phenotype.

### 2.8 The IGF2BP3^K76^‐Lactylation/PCK2/SAM Axis Facilitates NRF2 Expression in Lenvatinib‐Resistant HCC

**Figure 7:**
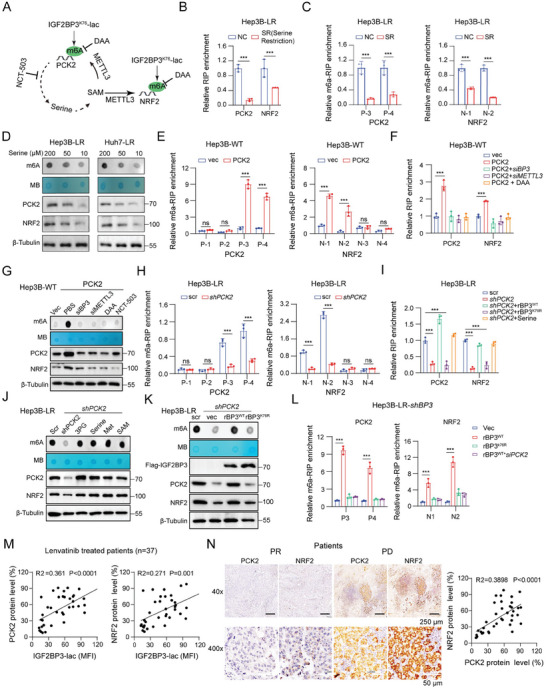
The IGF2BP3^K76^lac/PCK2/SAM axis facilitates NRF2 expression in lenvatinib‐resistant HCC. A) The diagram illustrates how IGF2BP3^K76^‐lac‐PCK2 orchestrates NRF2 upregulation via SAM‐dependent m6A modifications. B) RIP enrichment of PCK2 and NRF2 mRNA after serine restriction in Hep3B‐LR cells. C) MeRIP‒qPCR analysis of specific m6A motif enrichment in PCK2 and NRF2 mRNAs postserine restriction (SR) in Hep3B‐LR cells. D) RNA m6A dot blot assays and immunoblotting for PCK2 and NRF2 in lenvatinib‐resistant cells under serine restriction. Methylene blue staining and β‐tubulin were used as loading controls. E) MeRIP‒qPCR analysis of specific m6A motif enrichment of PCK2 and NRF2 mRNAs after PCK2 overexpression (OE) in Hep3B‐WT cells. (F) RIP analysis revealed enrichment of PCK2 and NRF2 mRNAs in Hep3B‐WT cells transfected with PCK2 OE following the inhibition of METTL3 or IGF2BP3 (siBP3) or treatment with 3‐deazaadenosine (DAA) (50 × 10^−6^ m). G) RNA m6A dot blot assays and Western blot analysis of PCK2 and NRF2 in Hep3B‐WT cells transfected with PCK2 OE following IGF2BP3 or METTL3 inhibition or treated with PBS, DAA (50 × 10^−6^ m) or NCT‐503 (20 × 10^−6^ m). Methylene blue (MB) staining and β‐tubulin were used as loading controls. (H) MeRIP‒qPCR analysis of m6A motif enrichment in PCK2 and NRF2 mRNAs after PCK2 inhibition in Hep3B‐LR cells. I) RIP analysis of PCK2 and NRF2 mRNAs in Hep3B‐LR cells with PCK2 inhibition rescued with rBP3^WT^ (rIGF2BP3^WT^), rBP3^K76R^ (rIGF2BP3^K76R^), or serine. J) RNA m6A dot blot assays and Western blot analysis of PCK2 and NRF2 in Hep3B‐LR cells with PCK2 inhibition treated with 3PG (0.75 × 10^−3^ m), serine (400 × 10^−6^ m), methionine (100 × 10^−6^ m), or SAM (50 × 10^−6^ m). Methylene blue staining and β‐tubulin were used as loading controls. K) RNA m6A dot blot assays and Western blot analysis of PCK2 and NRF2 in Hep3B‐LR cells with PCK2 inhibition and transfected with rBP3^WT^ or rBP3^K76R^. Methylene blue staining and β‐tubulin were used as loading controls. L) MeRIP‒qPCR analysis of m6A motif enrichment in PCK2 and NRF2 mRNAs in Hep3B‐LR‐shBP3 cells transfected with vec, rBP3^WT^, rBP3^K76R^, or rBP3^WT^+siPCK2. M) Correlation analysis between the mean fluorescence intensity (MFI) of Klac staining of IGF2BP3 (IGF2BP3‐lac) and PCK2 or NRF2 protein expression in HCC patients treated with lenvatinib (*n* = 37). N) Representative immunohistochemical images depicting PCK2 and NRF2 expression in patients with PR and PD‐related HCC after lenvatinib treatment (scale bar: black, 250 µm; white, 50 µm). Correlation between PCK2 and NRF2 protein expression in HCC patients treated with lenvatinib (*n* = 37). Statistical significance was assessed using two‐tailed unpaired Student's *t*‐test or one‐way analysis of variance (ANOVA). Each bar in the graph represents the mean ± SD (*n* = 3). Statistical notations: *p < 0.05, **p < 0.01, ***p < 0.001.

We validated the regulatory role of IGF2BP3^K76^‐lactylation on PCK2 and NRF2 through m6A modifications (Figure 4 and Figures S4, and S5, Supporting Information). IGF2BP3K76‐lactylation‐PCK2 facilitated SAM synthesis, promoting m6A modifications by upregulating serine synthesis (Figure 6G). Consequently, SAM produced by IGF2BP3^K76^‐lactylation‐PCK2 may further enhance the expression of both PCK2 and NRF2 (**Figure** **7**A). Under serine‐restricted conditions, the interaction between IGF2BP3 and PCK2/NRF2 mRNAs, as well as the m6A modifications on PCK2 and NRF2 mRNAs, decreased significantly. (Figure 7B,C and Figure S10A,B, Supporting Information). This led to a reduction in global m6A modifications and decreased expression of PCK2 and NRF2 in lenvatinib‐resistant cells (Figure 7D). Thus, IGF2BP3^K76^‐lactylation, PCK2, and SAM may form a positive feedback loop, enhancing the expression of PCK2 and NRF2 and increasing antioxidant capacity.

Our investigations revealed that PCK2 OE significantly increased m6A abundance in PCK2 and NRF2 mRNAs (Figure 7E and Figure S10C, Supporting Information). Furthermore, PCK2 OE markedly increased IGF2BP3 binding to NRF2 or PCK2 mRNA, increasing global m6A modifications and NRF2 protein expression, whereas these effects were counteracted by METTL3/IGF2BP3 knockdown or DAA (an m6A inhibitor) treatment (Figure 7F,G, and Figure S10D,E, Supporting Information). In contrast, PCK2 KD led to a marked reduction in m6A levels in PCK2 and NRF2 mRNAs in Hep3B/Huh7‐LR cells (Figure 7H and Figure S10F, Supporting Information), as did PCK2 binding to IGF2BP3 (Figure 7I). Notably, the addition of serine or overexpression of IGF2BP3^WT^, but not IGF2BP3^K76R^, completely restored binding to IGF2BP3 (Figure 7I and Figure S10G, Supporting Information). Furthermore, supplementation with SAM and related metabolites, including serine, glycine, and 3PG, effectively restored NRF2 expression in PCK2‐KD cells (Figure 7J and Figure S10H, Supporting Information). The overexpression of IGF2BP3^WT^, but not lactylation‐deficient IGF2BP3^K76R^, significantly restored PCK2 and NRF2 expression (Figure 7K and Figure S10I, Supporting Information) and significantly increased m6A levels in PCK2 and NRF2 mRNAs. However, IGF2BP3^WT^ combined with siPCK2 did not increase the m6A level in PCK2 or NRF2 mRNA (Figure 7L). These findings indicate that the lactylation of IGF2BP3 promotes m6A modification of PCK2 and NRF2 in a PCK2‐dependent manner. Additionally, we found that NRF2 knockdown did not affect PCK2 levels in lenvatinib‐resistant HCC cells (Figure S10J, Supporting Information). A favorable association between IGF2BP3^K76^‐lactylation and the expression of PCK2 or NRF2 was evident in specimens treated with lenvatinib (Figure 7M). A positive correlation between PCK2 and NRF2 protein levels was evident in patients receiving lenvatinib treatment (Figure 7N), as well as in the mRNA expression data from the LIMORE and TCGA (Figure S10K,L, Supporting Information). These findings underscore a feedback mechanism involving IGF2BP3^K76^‐lactylation and PCK2‐serine‐SAM that drive PCK2 and NRF2 expression, thereby increasing the antioxidant capacity of lenvatinib‐resistant HCC.

### 2.9 High IGF2BP3 Lactylation Levels are Correlated with Poor Therapeutic Responses to Lenvatinib, and Targeting IGF2BP3 Restores the Lenvatinib Response in HCC

**Figure 8:**
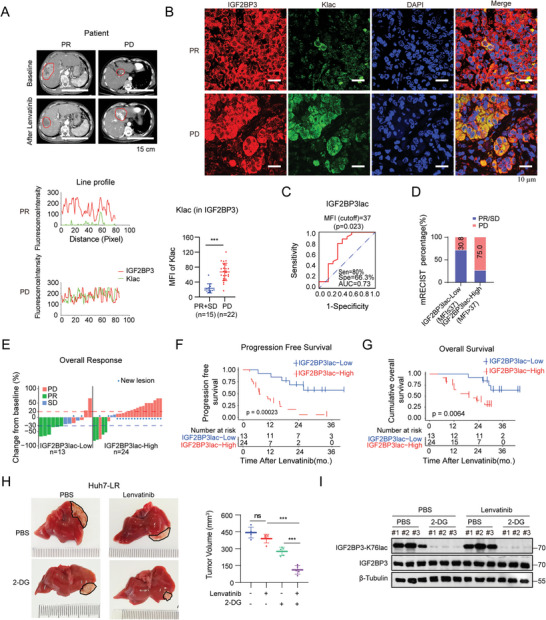
High IGF2BP3 lactylation levels correlate with poor therapeutic responses to lenvatinib, and targeting IGF2BP3 restores the lenvatinib response in HCC. A) CT images of HCC patients demonstrating partial response (PR) and progressive disease (PD) in response to lenvatinib treatment. Scale bar: 15 cm. B) Immunofluorescence staining of representative lenvatinib‐sensitive (PR+SD, *n* = 15) and lenvatinib‐resistant (PD, *n* = 22) patient samples. Confocal microscopy images showing colocalization of IGF2BP3 (red) and pan‐Klac (green) with nuclear staining by DAPI (blue) (scale bar, 10 µm). For colocalization analysis, ImageJ was used, and the mean fluorescence intensity (MFI) of Klac staining of IGF2BP3 was statistically analyzed via two‐tailed paired *t* test. C) AUC curves for predicting the response to lenvatinib via the mean fluorescence intensity (MFI) of Klac staining of IGF2BP3 (IGF2BP3lac) in posttreatment tumor samples with modified RECIST criteria. D,E) Evaluation of lenvatinib response in the Tongji Hospital cohort based on modified RECIST criteria (D). Waterfall plots displaying intrahepatic lesion responses in subgroups with high (*n* = 24) and low (*n* = 13) IGF2BP3lac levels treated with lenvatinib (E). F,G) Kaplan‒Meier survival curves depicting progression‐free survival (PFS) F) and overall survival (OS) G) relative to IGF2BP3lac expression were generated using SPSS 25.0. H) Representative images of the Huh7‐LR cell‐derived orthotopic HCC model at the endpoint (*n* = 5 per group). The mice were intraperitoneally injected with 100 mg k^−1^g 2‐DG every day for two weeks in combination with the oral administration of lenvatinib at 10 mg k^−1^g. I) Western blot analysis of IGF2BP3 lactylation levels in the Huh7‐LR cell‐derived orthotopic HCC model treated with 2‐DG alone or in combination with lenvatinib. Statistical significance was assessed via two‐tailed unpaired Student's t test or one‐way analysis of variance (ANOVA). Each bar represents the mean ± SD. Statistical notations: *p < 0.05, **p < 0.01, ***p < 0.001.

To determine the pivotal role of IGF2BP3lac in lenvatinib resistance, we conducted a detailed analysis of IGF2BP3lac levels in posttreatment surgical HCC samples (*n* = 37). Using the modified Response Evaluation Criteria in Solid Tumors (mRECIST), we observed that patients with low IGF2BP3lac levels responded better to lenvatinib, as evidenced by CT or MRI scans, than those with high IGF2BP3lac levels (**Figure** **8**A,B). We evaluated the potential of IGF2BP3lac as a biomarker for lenvatinib response using a posttreatment dataset. Receiver operating characteristic (ROC) curve analysis revealed an area under the curve (AUC) of 0.73 with an optimal cutoff of 37 (IGF2BP3lac, MFI = 37) based on the mRECIST criteria (Figure 8C), suggesting that IGF2BP3lac may function as a response indicator to lenvatinib. We classified the staining intensities as high for IGF2BP3lac (MFI≥37) or low for IGF2BP3lac (MFI< 37). Specifically, 9 of the 13 patients (69.2%) in the low‐IGF2BP3lac group achieved an objective response, which was significantly greater than the 6/24 (25.0%) in the high‐IGF2BP3lac group (p < 0.05) (Figure 8D). Notably, only four cases of disease progression were observed in the low‐IGF2BP3lac group, whereas the majority were in the high ‐IGF2BP3lac group (Figure 8E). Moreover, high IGF2BP3lac levels were associated with poorer progression‐free and overall survival in the Tongji hospital cohort (Figure 8F,G).

We sought to translate these findings into a therapeutic approach to reverse lenvatinib resistance. To evaluate the impact of glycolysis inhibition on lenvatinib sensitivity in vivo, we treated an orthotopic HCC model with the glycolysis inhibitor 2‐DG. The results revealed that 2‐DG treatment significantly increased lenvatinib sensitivity (Figure 8H) and reduced the levels of IGF2BP3lac in vivo (Figure 8I). We further evaluated the use of siIGF2BP3‐loaded liposomes (LNPs, lipid nanoparticles) for targeted delivery in an orthotopic xenograft model via the tail vein (Figure S11A, Supporting Information). The liposomes, optimized for high siRNA loading efficiency and liver targeting, exhibited greater than 90% packing efficiency and a uniform spherical shape with an average diameter of ≈100 nm, as observed via transmission electron microscopy (TEM) images (Figure S11C,D, Supporting Information). Continuous treatment with siRNA‐loaded liposomes (si‐LNPs) did not significantly affect liver toxicity markers, suggesting minimal liver toxicity and general toxicity (Figure S11E, Supporting Information). In vivo, liposomes predominantly accumulate in the liver and gradually decrease over time. siIGF2BP3‐loaded liposomes significantly suppressed IGF2BP3 expression, reaching the lowest levels on Day 3 post‐administration and returning to normal levels by Day 6 (Figure S11F,G, Supporting Information). Treatment with siIGF2BP3‐loaded liposomes every 3 days for two weeks combined with lenvatinib significantly reduced the liver tumor burden (Figure S11G, Supporting Information). These results confirm that high IGF2BP3lac levels correlate with poor therapeutic responses to lenvatinib and that targeting IGF2BP3 can effectively restore lenvatinib sensitivity in HCC.

## 3 Discussion

Emerging evidence has established a robust connection between dysregulated metabolism and epigenetic remodeling, significantly impacting tumorigenesis, progression, and chemoresistance.^[^ ^19^, ^27^, ^29^ ^]^ However, the precise mechanisms by which fluctuations in metabolite levels influence epigenetic changes and subsequent gene expression remain elusive. Our study addresses this gap by demonstrating that direct lactylation of IGF2BP3 is crucial for driving serine metabolism and maintaining redox homeostasis in lenvatinib‐resistant HCC. This process is mediated by the expression of key genes such as PCK2 and NRF2. Mechanistically, IGF2BP3 lactylation (IGF2BP3lac) enhances PCK2‐mediated gluconeogenesis, channeling carbon fluxes into serine and one‐carbon unit synthesis and sustaining elevated SAM and GSH levels. Increased SAM concentrations potentiate RNA m6A methylation through the methyltransferase METTL3. Lactylated IGF2BP3 subsequently binds to m6A sites on PCK2 and NRF2 mRNAs, stabilizing and promoting translation. This mechanism elucidates a novel regulatory axis of IGF2BP3lac‐PCK2 in lenvatinib‐resistant HCC, highlighting the intricate interplay between metabolic reprogramming and epigenetic modifications. Our study also suggests that IGF2BP3lac expression in HCC specimens may predict treatment response and that targeting IGF2BP3 or PCK2 could enhance lenvatinib efficacy.

Our study revealed that activated glycolysis and elevated lactate levels in lenvatinib‐resistant cells induced the lactylation of IGF2BP3 through nonhistone lysine lactylation. Lactate, a key metabolite in PTM, has been identified as a principal factor in lactylation, influencing gene expression through the lactylation of histones and other proteins.^[^ ^30^ ^]^ The high lactate production in lenvatinib‐resistant cells and elevated protein lactylation in lenvatinib‐resistant patients led us to hypothesize that protein lactylation may be a novel mechanism contributing to lenvatinib resistance. In this study, we identified, for the first time, that lactylation at the K76 site of IGF2BP3 is crucial for enhancing its capacity to capture target RNA. This novel finding elucidates a key mechanism by which IGF2BP3 interacts with specific RNA molecules, offering insights into the molecular dynamics of RNA‒protein interactions in the context of cellular processes. This finding aligns with recent research indicating the role of Klac in enhancing METTL3 function.^[^ ^23^ ^]^

PEPCK (PCK1 or PCK2), which is traditionally associated with gluconeogenesis, also regulates TCA cycle flux and increases glucose and pyruvate utilization for anabolic metabolism.^[^ ^28a^ ^]^ PCK2 is downregulated in human HCC.^[^ ^31^ ^]^ However, PCK2 activation is critical for cancer cell adaptation to environmental stresses such as glucose depletion, providing a metabolic advantage under such conditions. Reports indicate that sorafenib exposure triggers the upregulation of PCK2 in cells and clinical subjects, promoting metabolic reprogramming and sorafenib resistance.^[^ ^32^ ^]^ PCK2 upregulation can be induced by stressors, including chemotherapeutic agents such as gemcitabine. It provides intermediate sources essential for cell growth, including NADPH.^[^ ^33^ ^]^ NADPH is vital for biosynthetic reactions and maintaining redox balance, protecting against ROS toxicity, and facilitating GSH regeneration. Moreover, under chemically induced endoplasmic reticulum (ER) stress or amino acid deprivation, ATF4 activates PCK2, facilitating cancer cell adaptation to stress.^[^ ^34^ ^]^ Similarly, in human HCC cells (Hep3B/Huh7) and patient samples, lenvatinib treatment markedly induced PCK2 expression. PCK2 upregulation leads to gluconeogenesis, which can stabilize the nutrient supply under low‐glucose conditions, favoring the synthesis of critical metabolic intermediates essential for tumor growth.^[^ ^28^, ^35^ ^]^ These findings suggest that the role of PCK2 in enhancing energy efficiency and redox balance is key in resistance to multiple tyrosine kinase inhibitors.^[^ ^32^ ^]^ This metabolic flexibility highlights the importance of PCK2 in the adaptation and survival of cancer cells under adverse conditions.

Metabolic deregulation is a hallmark of cancer, with alterations, such as activated glycolysis and enhanced cholesterol biosynthesis, which are reported to affect lenvatinib treatment.^[^ ^36^ ^]^ Although therapy‐induced metabolic modifications in cancer cells have been the focus of limelight, the role of metabolic reprogramming in lenvatinib response in HCC remains unclear. Our work reveals a survival mechanism activated by lenvatinib in HCC cells that contributes to acquired resistance. Elevated IGF2BP3lac‐PCK2 in lenvatinib‐resistant cells redirects glucose toward serine anabolism. Serine metabolism, often altered in cancer, facilitates de novo synthesis of biomacromolecules and GSH/NADPH, driving cancer cell proliferation and drug resistance.^[^ ^13^, ^37^ ^]^ Our results indicate that IGF2BP3lac drives PCK2‐mediated substrate conversion (3‐PG) catalyzed by PHGDH into serine, supporting antioxidation reactions and biomass synthesis. This process facilitates antioxidant reactions and biomass synthesis. Corroborating our results, several studies indicate that TKI trigger ROS production and cell death signaling, while TKI‐resistant cells activate enzyme‐independent pathways to generate GSH/NADPH for ROS scavenging.^[^ ^25^, ^38^ ^]^ Consistent with our results, studies have revealed that serine synthesis inhibition abolished the mass balance in antioxidant capacity in TKI‐resistant cells.^[^ ^25^, ^37^ ^]^ This finding underscores the role of IGF2BP3lac in maintaining a balanced redox state derived from the carbon flux in gluconeogenesis.

SAM, generated during one‐carbon metabolism, serves as a universal methyl donor for DNA, histone, and RNA methylation.^[^ ^27^ ^]^ Our findings suggest that IGF2BP3lac modulates SAM production by channeling gluconeogenic intermediates such as 3PG into serine synthesis. Employing a sophisticated approach, we have, for the first time, substantiated the modulation of m6A levels by providing substrates to METTL3. PEPCK drives the accumulation of 3PG, a precursor of gluconeogenesis, ultimately leading to enhanced SAM biosynthesis.^[^ ^14^ ^]^ M6A methylation, dependent on SAM concentrations, is a dynamic RNA modification implicated in TKI resistance.^[^ ^39^ ^]^ Our study highlights a positive correlation between IGF2BP3lac and m6A levels in lenvatinib‐resistant cells. We propose that METTL3, responsible for N6‐RNA methylation, modulates RNA susceptibility to SAM in lenvatinib‐resistant cells. As pointed out in a previous study that PEPCK‐mediated metabolic remodeling contributes to histone methylation, which regulates cancer progression,^[^ ^14^ ^]^ we first pointed out that IGF2BP3lac mediated PCK2 upregulation promotes the whole levels of m6A‐modification and the anti‐TKI drug phenotype. This underscores the reciprocal regulation of epigenetic and metabolic remodeling.

Furthermore, our findings establish the critical role of IGF2BP3lac‐PCK2 in mediating serine metabolism and SAM generation, which forms a positive feedback loop leading to the upregulation of PCK2 and NRF2 expression, ultimately increasing antioxidant capacity. Reactive oxygen species (ROS) generation is a double‐edged sword in cancer therapy. Various antitumor therapies, including chemotherapy,^[^ ^40^ ^]^ TKIs,^[^ ^25^, ^41^ ^]^ and immunotherapy,^[^ ^42^ ^]^ induce ROS production, activating death signaling cascades in cancer cells.^[^ ^43^ ^]^ While they also prompt the activation of antioxidant defense mechanisms, potentially conferring resistance to therapies like Lenvatinib.^[^ ^18^ ^]^ Strong correlations between ROS and EGFR‐TKIs resistance have been demonstrated in lung cancer.^[^ ^44^ ^]^ Lenvatinib can induce hypoxia within HCC tissues.^[^ ^45^ ^]^ In hypoxic tumor environments, excessive ROS production prompts tumor cells to enhance antioxidant activity to maintain redox balance.^[^ ^46^ ^]^ Lenvatinib exacerbates oxidative stress by inducing ROS,^[^ ^47^ ^]^ but tumor cells counteract this by activating pathways such as NRF2‐mediated antioxidant gene expression.^[^ ^18^ ^]^ Our previous work demonstrated that LINC01607 induces lenvatinib resistance through the p62‐Keap1‐NRF2 axis.^[^ ^18^ ^]^ By binding to antioxidant response elements (AREs), NRF2 facilitates the transcription of antioxidant defense genes such as HO‐1, NQO1, and GPX4, thereby eliminating ROS and safeguarding cancer cells.^[^ ^48^ ^]^ In lenvatinib‐resistant HCC, we identified a regulatory mechanism in which IGF2BP3lac‐PCK2 modulates NRF2 expression, promoting enhanced ROS clearance.

Given the pivotal role of the IGF2BP3lac‐PCK2 axis in lenvatinib‐resistant patients, we believe that targeting this axis could represent a novel intervention strategy for lenvatinib‐resistant HCC. Consequently, targeting the IGF2BP3lac‐PCK2 axis represents a novel therapeutic strategy to overcome lenvatinib resistance in HCC. Our work not only elucidates the molecular mechanisms underlying lenvatinib resistance but also demonstrates that targeting IGF2BP3 with siRNA‐loaded liposomes can resensitize resistant HCC cells to lenvatinib in vivo, suggesting a promising approach for clinical intervention. Additionally, small‐molecule inhibitors targeting the IGF2BP3^K76^ lactylation modification site hold significant promise for overcoming resistance to lenvatinib. Moreover, as lactylation of the IGF2BP3^K76^ site occurs exclusively in the context of lenvatinib resistance, small‐molecule inhibitors targeting the IGF2BP3^K76^‐lactylation site hold significant promise for overcoming lenvatinib resistance.

## 4 Conclusion

In conclusion, high levels of IGF2BP3lac are pivotal for promoting m6A modification by driving serine synthesis and SAM generation in lenvatinib resistance. Lactylated IGF2BP3 enhances its role as an m6A reader, stabilizing and facilitating the translation of PCK2 and NRF2 mRNAs. This process cooperatively promotes lenvatinib resistance in HCC. Our findings provide a novel perspective on the interplay between IGF2BP3lac‐PCK2‐mediated serine metabolism and m6A methylation in lenvatinib resistance and suggest that metabolic fluctuations are closely linked to epigenetic dysregulation. Thus, targeting these pathways to increase SAM availability and suppress IGF2BP3lac or PCK2 offers a potential therapeutic strategy to overcome lenvatinib resistance in HCC.

## 5 Experimental Section

### Clinical Sample Acquisition

This investigation, conforming to the Helsinki Declaration, received approval from the Institutional Review Board at Tongji Hospital's Hepatic Surgery Center, Huazhong University of Science and Technology. Informed consent was duly acquired from all subjects. The study included 37 HCC patients who underwent lenvatinib therapy before surgical intervention between October 2018 and December 2022. Efficacy of lenvatinib was assessed by the modified Response Evaluation Criteria in Solid Tumors (mRECIST1.1).^[^ ^49^ ^]^ Treatment outcomes were dichotomized as lenvatinib‐responsive (encompassing complete response [CR], partial response [PR], and stable disease [S] or lenvatinib‐resistant (progressive disease [PD]). Comprehensive radiological images before and after therapy were available for all participants. Tumor samples and adjacent nontumor tissues were formalin‐fixed and paraffin‐embedded. Subsequent immunohistochemistry (IHC) and immunofluorescence (IF) staining facilitated the examination of IGF2BP3lac and PCK2/NRF2 expression patterns. Overall survival (OS) was quantified as the duration from the commencement of lenvatinib treatment to the date of mortality.

### Ethics Approval and Consent to Participate

All procedures were approved by the Ethics Committee of Tongji Hospital (TJ‐IRB20230863) and were conducted in accordance with the Declaration of Helsinki and Istanbul. Written informed consent was obtained from all the patients. All animal studies were performed following a protocol approved by the Institutional Ethics Committee of Tongji Hospital, according to Animal Research Reporting In Vivo Experiments (TJH‐202212001).

### Cell Culture

The HCC cell lines Hep3B (ATCC HB‐8064), Huh7, and Hepa1‐6, sourced from the Shanghai Institute of Cell Biology, Chinese Academy of Sciences, were cultured in MEM (Hep3B) and DMEM (Huh7 and Hepa1‐6) (Hyclone, UT, USA) supplemented with 10% fetal bovine serum (Gibco) and 1% penicillin‐streptomycin. Cultivation was at 37 °C in a 5% CO2 atmosphere. Regular Mycoplasma testing confirmed the authenticity of these cell lines.

### Establishment of Acquired Lenvatinib‐Resistant HCC Cell Models

Lenvatinib resistant cell lines Hep3B‐LR, Huh7‐LR was previously constructed and maintained at our laboratory.^[^ ^18^ ^]^ After establishment, the resistant cell lines were continuously cultured in the presence of (5–10) × 10^−6^ m lenvatinib. As for lenvatinib resistant mouse model (Hepa1‐6 LR), the procedure was determined by referring to previous studies.^[^ ^50^ ^]^ Hep1‐6 cells (5×10^6^ cells per mouse) were subcutaneously inoculated into the right posterior flanks of male C57BL/6 mice (four weeks). Treatment with lenvatinib (10 mg k^−1^g day^−1^) was commenced once the tumors reached a volume of 100 mm^3^. After a 28‐day treatment period, the mice were euthanized. The largest tumor was dissected into 1 mm^3^ fragments, which were subsequently subcutaneously implanted into the next generation of four‐week‐old male C57BL/6 mice. This procedure was repeated across three generations, yielding a stable lenvatinib‐resistant Hep1‐6 cell line.

### Seahorse Assay

An XF24 Extracellular Flux Analyzer (Seahorse Bioscience, North Billerica, MA, USA) was utilized to measure the extracellular acidification rate (ECAR) in both parental and Lenvatinib‐resistant cell lines. Cells were plated at a density of 1×10^4^ cells per well in a 96‐well Seahorse XF Cell Culture Microplate (Agilent, USA) and incubated overnight. Following a gentle wash with PBS, the cells were incubated for one hour at 37 °C in Seahorse incubation medium containing 1 × 10^−6^ m glucose and 2 × 10^−3^ m L‐glutamine. To ensure accurate extracellular pH measurements, the cells were maintained in a CO2‐free incubator. ECAR was measured at baseline and after sequential injections of rotenone/antimycin A (Rot/AA, 0.5 × 10^−6^ m) and 2‐DG (50 × 10^−3^ m). The ECAR values are presented as the mean ± SD. Measurements were conducted according to the Seahorse XF Glycolysis Rate Test Assay manual (103344‐100; Agilent Technologies, North Billerica, MA). After each measurement, the protein concentration in the living cells within each well was determined using the Pierce Rapid Gold BCA Protein Assay for subsequent normalization.

### Animal Experiments

Four‐week‐old male wild‐type C57BL/6J and BALB/c nude mice were purchased from Gem Pharmatech Co. Ltd. (Jiangsu, China). Ethics approval was obtained from the ethics committee of Tongji Hospital according to the Animal Research Reporting In Vivo Experiments. All animals received humane care according to the criteria outlined in the “Guide for the Care and Use of Laboratory Animals.” Mice were euthanized with CO2 when their tumor burden and overall health status met established criteria. The tumor volume was measured using color Doppler ultrasonography. The formula used to calculate the volume (*V*) was as follows: volume (*V*) = length (*a*) × width (*b*)^2^ × (π/6), as previously reported.^[^ ^51^ ^]^

### Hydrodynamic Injection Mouse Model

Six‐week‐old male C57BL/6J mice were procured for this study. Hydrodynamic injection was performed according to previously established protocols. This involved diluting A mixture of 20 µg N‐Ras^G12V^, 20 µg myr‐AKT1, 4 µg Sleeping Beauty (SB) transposase, and various plasmids (30 µg each of PT2‐shScramble, PT2‐shIGF2BP3, PT2‐shIGF2BP3 + PT3‐rIGF2BP3^WT^, and PT2‐shIGF2BP3 + PT3‐rIGF2BP3^K76R^) in 2 mL PBS. The solution was rapidly injected into the lateral tail vein within 2 seconds. After a 4‐week period, lenvatinib treatment was commenced, involving a daily oral administration of 10 mg k^−1^ g in PBS for 14 days. After treatment, mice with tumor development were euthanized, and liver tissues were harvested for analysis.

### Orthotopic Xenograft Mouse Model and Liposomes Treatment

1×10^6^ Huh7‐LR cells in 20 µL PBS were injected into the left hepatic lobe of six‐week‐old male NCG mice. Oral lenvatinib treatment was initiated when the tumor size reached ≈100 mm^3^. siRNA‐loaded liposomes were formulated as per previously established methods.^[^ ^52^ ^]^ A lipid mixture (12.5 µL) was combined with 10 × 10^−3^ m sodium citrate, followed by vortex oscillation for 10 s. SiIGF2BP3 or siNC dissolved in citrate buffer was then mixed with the lipid at a 5:1 lipid/siRNA weight ratio. The mixture was subjected to ultrafiltration centrifugation to remove free siRNA and was subsequently diluted in PBS. Liposome‐siRNA characteristics, such as zeta potential, polydispersity, and hydrodynamic diameter, were measured using DLS (Malvern Zetasizer Nano‐ZS, UK). siRNA entrapment efficiency was assessed using the RiboGreen assay. The liposomes were further examined using TEM (Jeol, Japan) after staining with 2% phosphotungstic acid. The mice were divided into four groups: PBS, Liposome, PBS + lenvatinib, and Liposome + lenvatinib.

### In Vivo Biodistribution of the Liposomes

DiR was used to label the liposomes according to the manufacturer's instructions. The prepared liposome‐siRNA was intravenously injected into the tail vein of the mice after orthotopic liver tumors reached ≈100 mm^3^. Subsequently, the mice were anesthetized with isoflurane (Sigma‐Aldrich, St. Louis, MO, USA) and imaged using an in vivo imaging system (IVIS Lumina XR, SI Imaging, AZ, USA) (excitation: 745 nm, emission: 830 nm) at different time points (0 h, 1d, 3d, 6d). After the treatment period, the mice were sacrificed under CO2 and the tumors were analyzed.

### RNA Interference

Hep3B/Huh7‐LR cells were transfected with siRNA using InvitroRN Reagent (InvivoGene Biotechnology, Suzhou, China), according to the standard protocol. The siRNA sequences used are listed in Table S1 (Supporting Information).

### 4D Label‐Free Proteomics Analysis

Proteomic analysis of lactylation was conducted in collaboration with the Jingjie PTM BioLabs, Hangzhou, China. Proteins from both parental and lenvatinib‐resistant Hep3B cells were prepared by trypsin digestion, followed by centrifugation. The resulting tryptic peptides were resuspended in NETN buffer (0.5% NP‐40, 100 × 10^−3^ m NaCl, 50 × 10^−3^ m Tris–HCl, and 1 × 10^−3^ m EDTA, pH 8.0). Klac‐modified peptides were enriched overnight at 4 °C using PTM‐1404 antibody beads (PTM Bio), with gentle agitation. After enrichment, peptides were eluted and subsequently fractionated using a 300Extend C18 column (Agilent, Santa Clara, USA) for detailed liquid chromatography‐tandem mass spectrometry (LC‐MS/MS) analysis.

### RNA Extraction, Quantitative PCR, and Sequencing Analysis

RNA was isolated using the FastPure Cell/Tissue Total RNA Isolation Kit V2 (Vazyme), following the manufacturer's protocol. Subsequently, reverse transcription was performed using the HiScript II Q Select RT SuperMix (+gDNA wiper), according to the manufacturer's instructions. Quantitative PCR (qPCR) was performed using ChamQ Universal SYBR qPCR Master Mix. Gene expression levels were normalized to β‐actin levels in the respective samples. Independent assays were performed in triplicate for each sample. Primer sequences are detailed in Tables S2 and S3 (Supporting Information). After the assessment of RNA integrity, an mRNA library was generated and sequenced by Novogene Technology Co., Ltd. (Beijing, China). All next‐generation sequencing experiments were performed using an Illumina NovaSeq sequencer (Illumina). Differentially expressed genes were subsequently screened and identified using the criteria |log2FC|> 1 and FDR < 0.05.

### Methylated RNA Immunoprecipitation (MeRIP) qPCR Assay

Methylated RNA immunoprecipitation (MeRIP) assays were performed using the BersinBio MeRIP m6A Kit, according to the manufacturer's protocol. Initially, RNA from HCC cells was extracted and then fragmented for 5 min at 94 °C using a fragmentation buffer. This was followed by overnight incubation at 4 °C of the fragmented RNA with anti‐m6A antibody‐coated magnetic beads. The bead‐antibody complexes were washed and subsequently mixed with DNA‐free RNA. The eluted RNA was purified using QIAGEN RNA Extraction Solution and analyzed by quantitative PCR (qPCR). The MeRIP‐qPCR primers for the IGF2BP3‐targeted m6A site are listed in Table S4 (Supporting Information).

### RNA‐Immunoprecipitation Seq, RIP‐qPCR

RNA immunoprecipitation (RIP) was performed using the Magna RIP RNA‐Binding Protein Immunoprecipitation Kit (Merck Millipore) with an anti‐IGF2BP3 antibody. Following immunoprecipitation, RNA was purified and subjected to quantitative PCR (qPCR) analysis. For next‐generation sequencing (NGS), both input and RIP samples containing 150–200 ng of RNA were processed. NGS library preparation was carried out using an Illumina Kit with sequencing services provided by the SeqHealth Company, Wuhan, China.

### M6A Dot Blot Assay

RNA extraction was performed using TRIzol reagent (Takara), and mRNA was purified using the Dynabeads mRNA Purification Kit (Invitrogen). mRNA concentration and purity were assessed using NanoDrop 2000. For denaturation, mRNA (100–400 ng) was heated at 95 °C for 3 min and then immediately cooled on ice. Denatured mRNA was transferred onto a positively charged nylon membrane (GE Healthcare) and air‐dried for 5 min. The UV crosslinking was conducted at a wavelength of 1200 nm. The membrane was blocked in 5% nonfat milk in PBST, followed by overnight incubation at 4 °C with an anti‐m6A antibody (Abclonal). After incubation with an HRP‐conjugated anti‐rabbit IgG secondary antibody for 1 h at room temperature, visualization was achieved via enhanced chemiluminescence. Methylene blue staining (0.02%, Sigma‐Aldrich) was used to confirm the uniform mRNA deposition on the membrane.

### DNA Dot Blot

Genomic DNA extraction was performed using the TIANamp Genomic DNA Kit (TIANGEN Biotech, Beijing, China) according to the manufacturer's protocol. DNA was diluted in 0.1 M NaOH and denatured at 95 °C for 10 min before rapid cooling on ice. The following steps mirrored the m6A dot blot assay, replacing it with an anti‐5‐mC antibody.

### RNA Stability Assay

HCC cell lines (Hep3B‐LR/Huh7‐LR siNC or siIGF2BP3/siMETTL3) were seeded in 6‐well plates. The cells were treated with actinomycin D (5 µg mL^−1^, MCE) for 0, 3, 6, 9, and 12 h to inhibit transcription. At these time intervals, RNA was extracted and quantified using quantitative PCR (qPCR). The half‐life of mRNA was calculated using linear regression analysis based on RNA decay rates.

### M6A Motif Prediction and Synthesis of m6A Probes

The m6A motifs in PCK2 and NRF2 transcripts were predicted using the SRAMP prediction tool (http://www.cuilab.cn/sramp). Biotinylated RNA probes for PCK2 and NRF2, ≈20 nucleotides in length and encompassing predicted m6A motifs, were synthesized by Tsingke Biotechnology (Beijing, China). These probes were designed in three variants: methylated, unmethylated adenosine, and adenosine‐mutated forms.

### Biotin‐Labeled RNAs Pull‐Down

The Pierce Magnetic RNA‐Protein Pull‐Down Kit (Thermo Fisher Scientific) was used for the in vitro RNA pull‐down assays. Specifically, 50 pmol of biotinylated PCK2 and NRF2 RNA probes, with either methylated or unmethylated adenosine, was incubated with 2 mg of protein extract and 50 µL of pre‐washed streptavidin magnetic beads. The mixture was incubated at room temperature for 1 h, followed by three washes. The beads were boiled in SDS buffer for western blot analysis. The details of the RNA probe sequences are provided in Table S5 (Supporting Information).

### Polysome Profiling

Polysome profiling was performed as described in a previous study.^[^ ^53^ ^]^ Hep3B‐LR and shIGF2BP3 cells were treated with 100 µg mL^−1^ cycloheximide (CHX) for 10 min at 37 °C. After treatment, the cells were washed three times with ice‐cold CHX/PBS and then scraped and lysed on ice for 10 min using a lysis buffer containing 20 × 10^−3^ m Tris–HCl (pH 7.4), 10 × 10^−3^ m MgCl_2_, 300 × 10^−3^ m NaCl, 100 µg mL^−1^ CHX, 1% Triton X‐100, 1 × 10^−3^ m DTT, 0.5% sodium deoxycholate, 1× RNase inhibitor, and 1× EDTA‐free protease inhibitor cocktail. The lysate was centrifuged at 12000 × *g* for 10 min at 4 °C. The supernatant was then layered onto a 5–50% w/v sucrose gradient and centrifuged at 260 808 × *g* at 4 °C. This gradient was fractionated into 15 aliquots (0.5 mL) and analyzed by qPCR for PCK2 transcripts.

### Untargeted Metabolomics Analysis

Untargeted metabolomic profiling of Hep3B LR‐shIGF2BP3 cells transfected with either Flag‐rIGF2BP3^WT^ or Flag‐rIGF2BP3^K76R^ was conducted using MetWare (http://www.metware.cn/) on an AB Sciex QTRAP 6500 LC‐MS/MS platform.

### Metabolite Extraction and HLPC Analysis of L‐Serine and Glycine

Cell samples: Following a previously established protocol,^[^ ^54^ ^]^ Cells were harvested and washed twice with ice‐cold PBS. The cell pellet was resuspended in cold MeOH/H2O (80:20) at a ratio of 1×10^6^ cells/100 µl. The suspension was vigorously vortexed and subjected to a freeze‐thaw cycle in an ice bath for 10 min, followed by storage at −80 °C for 10 min. This freeze‐thaw process was repeated twice. The supernatant was separated by centrifugation at 12 000 × *g* for 10 min at 4 °C. A second extraction was performed on the pellet using an equivalent volume of extraction solvent, and the combined extracts were used for the HPLC analysis of L‐serine and glycine.

Tissue samples for metabolite extraction: 60 mg of powdered tissue was mixed with 100 µL of a chilled mixture of methanol, acetonitrile, and water in a 2:2:1 ratio (v/v/v). The mixture was vortexed and incubated on ice for 20 minutes. Post incubation, samples were centrifuged at 14 000 × *g* for 20 min at 4 °C. The resulting supernatant (100 µL) was analyzed using LC‐MS.

### ^13^C Labeled Glucose and Pyruvate Tracing

For ^13^C‐glucose labeling, the cells were cultured in MEM (no glucose, Procell, PM150462) supplemented with 5 × 10^−3^ m U‐[13C]‐d‐glucose (Cambridge Isotope Laboratories, Inc., Cat# CLM‐1396‐1) for a 12 h. For ^13^C‐pyruvate labeling, the cells were cultured in MEM (without Sodium Pyruvate and glucose, Procell, Cat#PM150462) supplemented with 1 × 10^−3^ m U‐[13C]‐pyruvate (Cambridge Isotope Laboratories, Inc., Cat#CLM‐2440‐0.1) for a 12 h. Subsequently, the cells were washed with cold PBS and metabolites were extracted using 80% cold methanol. The Shanghai Metabolome Institute (SMI)‐Wuhan conducted UHPLC‐MS analysis, and the abundance of metabolites was quantified relative to the total area of labeled and unlabeled carboxylic acid analyte/ amino analyte, following established protocols.

### Measurement of SAM Levels via Enzyme Linked Immunosorbent Assay ELISA

SAM concentrations in the cell samples were quantified using a human SAM detection kit (Cell Biolabs, Inc., Catalog Number: MET‐5152, USA) according to the manufacturer's instructions. The absorbance was read at 450 nm using a microplate reader to determine SAM levels. Further details on the testing procedure can be found on the manufacturer's official website.

### Glucose Uptake Assay

Glucose uptake in cells was assessed using the fluorescent glucose analog, 2‐NBDG (2‐(N‐(7‐nitrobenz‐2‐oxa‐1,3‐diaxol‐4‐yl) amino)‐2‐deoxyglucose) at a concentration of 20 × 10^−6^ m (Invitrogen). cells were incubated with 2‐NBDG for 1 h at 37 °C. Post‐incubation, glucose uptake was quantified by measuring the fluorescence intensity using flow cytometry (BD Biosciences).

### Quantification of L‐lactic Acid

L‐lactic acid concentrations were determined using a Lactic Acid assay kit (Nanjing Jiancheng Bioengineering Institute, A019‐2‐1) according to the manufacturer's protocol.

### Cell Viability Assay

Cells in the logarithmic growth phase were seeded in 96‐well plates at a density of 3000 cells/well. Varying concentrations of the drug were added to six replicate wells. After 48 h of incubation, 10 µL of CCK‐8 solution (Vazyme Biotech Co., Ltd, A311) was mixed with 90 µL of culture medium in each well, followed by incubation in darkness at 37 °C for 1 h. Absorbance was measured at 450 nm using a microplate reader. The half‐maximal inhibitory concentration (IC50) values were calculated using GraphPad Prism (version 8, GraphPad Software, San Diego, CA, USA).

### Reactive Oxidative Species (ROS) Measurement

To evaluate ROS production, cells were treated as specified, then stained with CellROX Green ROS detection solution (Invitrogen) for 30 min at 37 °C in darkness, following the manufacturer's instructions. The fluorescence intensity, indicative of ROS levels, was quantified using flow cytometry.

### Apoptosis Assay

An Annexin V‐FITC/PI Apoptosis kit (Nanjing Vazyme Biotechnology Company, China) was used to assess cell apoptosis. The proportion of apoptotic cells was evaluated using the CytoFLEX flow cytometer (Beckman Coulter). FlowJo (version10.8.1) software was used to analyze the flow cytometry data.

### GSH/GSSG Ratio, NADPH/NADP+ Ratio Quantification

Intracellular glutathione (GSH) levels and the GSH/GSSG ratio were measured using the GSH and GSSG Assay Kit (Biovision, K264), according to the manufacturer's protocol. The absorbance of GSH and GSSG was measured at 412 nm using a microplate reader. The intracellular NADPH/NADP+ ratio was quantified using the NADP+/NADPH Assay Kit (Beyotime, S0179) following the manufacturer's guidelines. The absorbance of NADP+ and NADPH was measured at 450 nm wavelength using a microplate reader.

### Western Blot and Co‐Immunoprecipitation

Following established protocols,^[^ ^55^ ^]^ cells were lysed using lysis buffer (Cell Signaling Technology), enhanced with complete ULTRA Tablets (Roche) and PhosphoStop (Roche) as protease and phosphatase inhibitors, respectively. Protein concentrations were determined using a BCA assay (Thermo Fisher Scientific). Equal amounts of protein were separated by SDS‐PAGE and transferred to a PVDF membrane (Millipore). The membrane was blocked with 5% nonfat milk in Tris‐buffered saline (pH 7.4) with 0.1% Tween‐20 and probed with the appropriate primary and HRP‐conjugated secondary antibodies. The detection was performed using FluorChem E (Cell Biosciences).

For co‐immunoprecipitation, cells were lysed in IP lysis buffer on ice. Lysates were first incubated with protein A/G magnetic beads (LinkedIn Biotechnology Co., Ltd., Shanghai, China) for for 2 h, followed by overnight immunoprecipitation at 4 °C with specific antibodies. After binding to protein G agarose beads for 1 h, the lysates were washed with IP lysis solution and rinsed thrice with wash buffer (300 × 10^−3^ m NaCl, 1.0 × 10^−3^ m EDTA, 25 × 10^−3^ m Tris–HCl, pH 7.4, 1.0% NP‐40). The complexes were released using 2× SDS‐PAGE loading solution and analyzed by Western blotting. Antibodies used in this study are listed in Table S6 (Supporting Information).

### Immunohistochemical Analysis

The immunohistochemical staining assay was performed as described previously.^[^ ^55^ ^]^ Paraffin‐embedded slides were subjected to hematoxylin and eosin (HE) staining before IHC analysis. For IHC staining, a DAB substrate kit (Zsbio Commerce Store) was used following the guidelines provided by the manufacturer. Antibodies used in this study are listed in Table S6 (Supporting Information).

### Immunofluorescence and Confocal Microscopy Imaging

Immunofluorescence experiments were performed according to a previously described protocol. Briefly, cells were cultured on glass‐bottom cell culture dishes for 12 h, fixed with 4% paraformaldehyde for 15 min at room temperature, and permeabilized with 0.5% Triton X‐100 for 5 min. After blocking, the slides were incubated with primary antibodies overnight at 4 °C in a humidified environment. After three washes, the slides were incubated with secondary antibodies for 1 h at room temperature in a humidified setting. Finally, the nuclei were counterstained with 40, 60‐diamidino‐2‐phenylindole (DAPI) for 5 min. The resulting signals were captured using a confocal laser‐scanning microscope (Olympus FV1000, Tokyo, Japan). The relative fluorescence intensity and colocalization were assessed and quantified using the FIJI (ImageJ) software.

Following deparaffinization and rehydration, heat‐induced antigen retrieval was performed using Tris‐EDTA buffer (pH 9.0). Samples were blocked with 5% bovine serum albumin (BSA) and stained with IGF2BP3 mouse antibody (Santa Cruz Animal Health) and anti‐L‐Lactyl Lysine Rabbit mAb (PTM BioLabs) at 1:200 dilution in 5% BSA each for overnight at 4 °C, followed by secondary antibodies were used: Alexa Fluor anti‐rabbit A488 (1:200 dilution in 5% BSA, Proteintech) for lactylation, and anti‐mouse A594 (1:200 dilution in 5% BSA, Jackson) for IGF2BP3 staining. IGF2BP3 staining was pseudocolored red, and lactylation staining was pseudocolored green. The nuclei were counterstained with DAPI. Immunofluorescence images were captured using a laser scanning confocal microscope (OLYMPUS IX83‐FV3000, Tokyo, Japan). Quantification of lactylation in IGF2BP3 was performed using the FIJI (ImageJ) software as follows: 600× resolution images were obtained for each section. The mean fluorescence intensity (MFI) value was computed by initially marking the colocalization regions, recording the MFI of lactylation in these regions, and subsequently dividing the integrated density by the area. Statistical analysis was conducted using paired *t*‐tests to compare the mean gray values between tumor samples and adjacent normal samples. Antibodies used in this study are listed in Table S6 (Supporting Information).

### Statistical Analysis

Data were analyzed using GraphPad Prism version 9.0 (GraphPad, San Diego, CA, USA). Quantitative results are presented as means ± SD (*n* = 3), as indicated in the figure legends. For comparisons between two groups, a two‐tailed unpaired or paired Student's *t*‐test was employed based on the specific context noted in the figure legends. Comparisons among multiple groups were performed using a one‐way ANOVA. Kaplan–Meier survival curves were analyzed using the log‐rank test. ROC curve and area under the curve analyses were applied to detect the optimal cutoff point that yielded the highest total accuracy for discriminating sensitive or resistant to Lenvatinib. A p‐value < 0.05 was deemed to indicate Statistical significance was set at tests, with significance levels denoted as follows: *p < 0.05, **p < 0.01, ***p < 0.001, and ns for nonsignificant differences. Error bars in the graphical representations correspond to the mean ± SD, as specified.

## Conflict of Interest

The authors declare no conflict of interest.

## Author Contributions

Y.L., J.Z. and Y.Z. contributed equally to this work. W.Z. conceived and designed the experiments. Y.L., J.Z., Y.Z., and J.Z. performed the experiments and analyzed the data. W.Z., Y.L., J.Z., and J.Z. wrote the manuscript. W.Z., H.L., and C.S. contributed to project administration and revised the paper. H.L., Y.X., Y.W., and Y.F. contributed to data generation and data analysis. W.L. participated in the experiment and reviewed the article during the revision process. All the authors have read and approved the final version of the manuscript.

## Supporting information



Supporting Information

Supplemental Table 1

## Data Availability

The data that support the findings of this study are available from the corresponding author upon reasonable request.
