# Supplementary material for: Lactylation‐Driven IGF2BP3‐Mediated Serine Metabolism Reprogramming and RNA m6A—Modification Promotes Lenvatinib Resistance in HCC
Source: Adv Sci (Weinh). 2024 Oct 25;11(46):2401399. doi: 10.1002/advs.202401399 (PMC11633555; doi:10.1002/advs.202401399)
Supplement: Supplementary file 1 — Supporting Information [file ADVS-11-2401399-s001.docx]

**Figure S1** Elevated glycolytic activity and heightened lactylation modifications in lenvatinib-resistant cell lines impede sensitivity to lenvatinib

(A) Determination of IC50 values in three lenvatinib-resistant cell lines and the corresponding parental cell lines using the CCK8 assay.

(B) Enrichment plots showing the glycolysis/gluconeogenesis pathways analyzed by gene set enrichment analysis (GSEA) of the GEO datasets for Hep3B-WT cells treated with lenvatinib (GSE198845).

(C and D) GSVA revealed notable upregulation of the glycolysis pathway in intrinsically resistant HCC cell lines based on Liver Cancer Model Repository (LIMORE) (C) and Cancer Cell Line Encyclopedia (CCLE) datasets (D).

(E) The ECAR profile was monitored in Hep3B-WT, Hep3B-LR, Huh7-WT and Huh7-LR cell lines using the seahorse assay.

(F) Total pan-Klac levels in Huh7-WT cells treated with lenvatinib over various months were measured using Western blotting.

(G) Assessment of pan-Klac levels in lenvatinib-sensitive or intrinsically resistant HCC cell lines using Western blotting. The IC50 values for lenvatinib in HCC cell lines were detected using the CCK8 assay.

(H) Western blot analysis of the pan-Klac levels in Hep3B-WT or Huh7-WT cells treated with Nala (25 mM) at the indicated times.

(I) Western blot analysis of the pan-Klac levels in Huh7-LR cells treated with 2-DG (10 mM) at the indicated times.

(J) Determination of IC50 values in Huh7-LR cells cultured with 2-DG (10 mM) for 48 hours using the CCK8 assay.

(K) Analysis of apoptosis in Hep3B-LR or Huh7-LR cells treated with lenvatinib (40 μM) alone or in combination with 2-DG (10 mM) using flow cytometry with Annexin V staining.

(L) siRNA screening revealed that IGF2BP3 lactylation may promote lenvatinib resistance in Huu7-LR cell lines.

Statistical analyses were performed using two-tailed unpaired Student’s t-test. Each bar in the graph represents the mean ± SD (n = 3). Statistical notation: ns (not significant), **P* < 0.05, ***P* < 0.01, ****P* < 0.001.

**
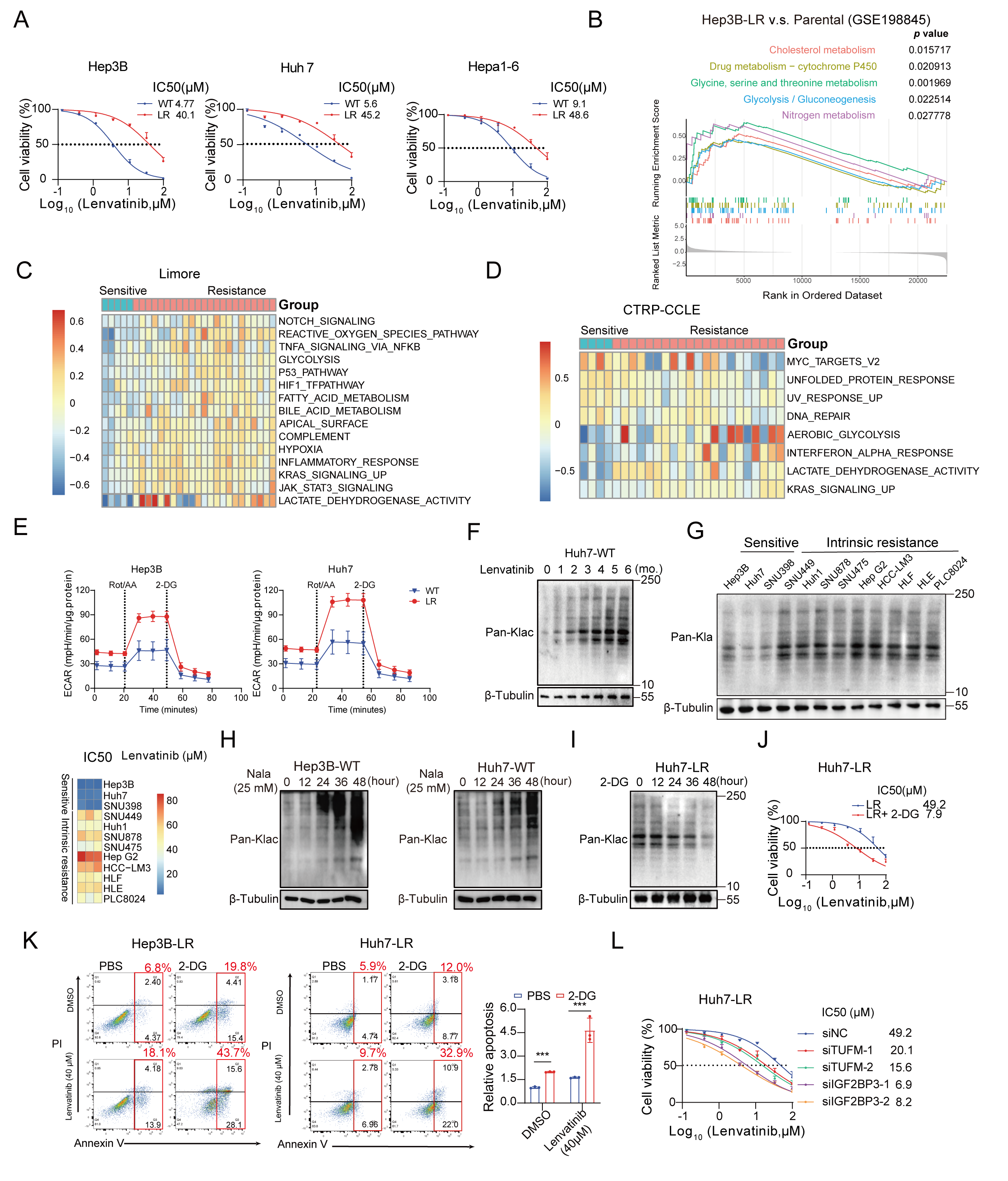
Figure S1**

**Figure S2** Increased IGF2BP3^K76^ lactylation is responsible for resistance to lenvatinib

(A) IC50 determination and Western blot analysis of IGF2BP3 levels in Huh7-LR cells transduced with shBP3.

(B) IC50 assay and Western blot analysis of IGF2BP3 lactylation levels in Huh7-LR cells transduced with shBP3 plus vec, rBP3^WT^, or the shRNA-resistant mutation-rBP3^K76R^ (rIGF2BP3^K76R^) plasmid.

(C) Evaluation of apoptosis rates in Huh7-LR cells transduced with shBP3 complemented with vectors encoding vec, rBP3^WT^, or the rBP3^K76R^ plasmid.

Statistical analysis was performed using one-way analysis of variance (ANOVA). Each bar represents the mean ± SD (n = 3). Statistical notations: **P* < 0.05, ***P* < 0.01, ****P* < 0.001.


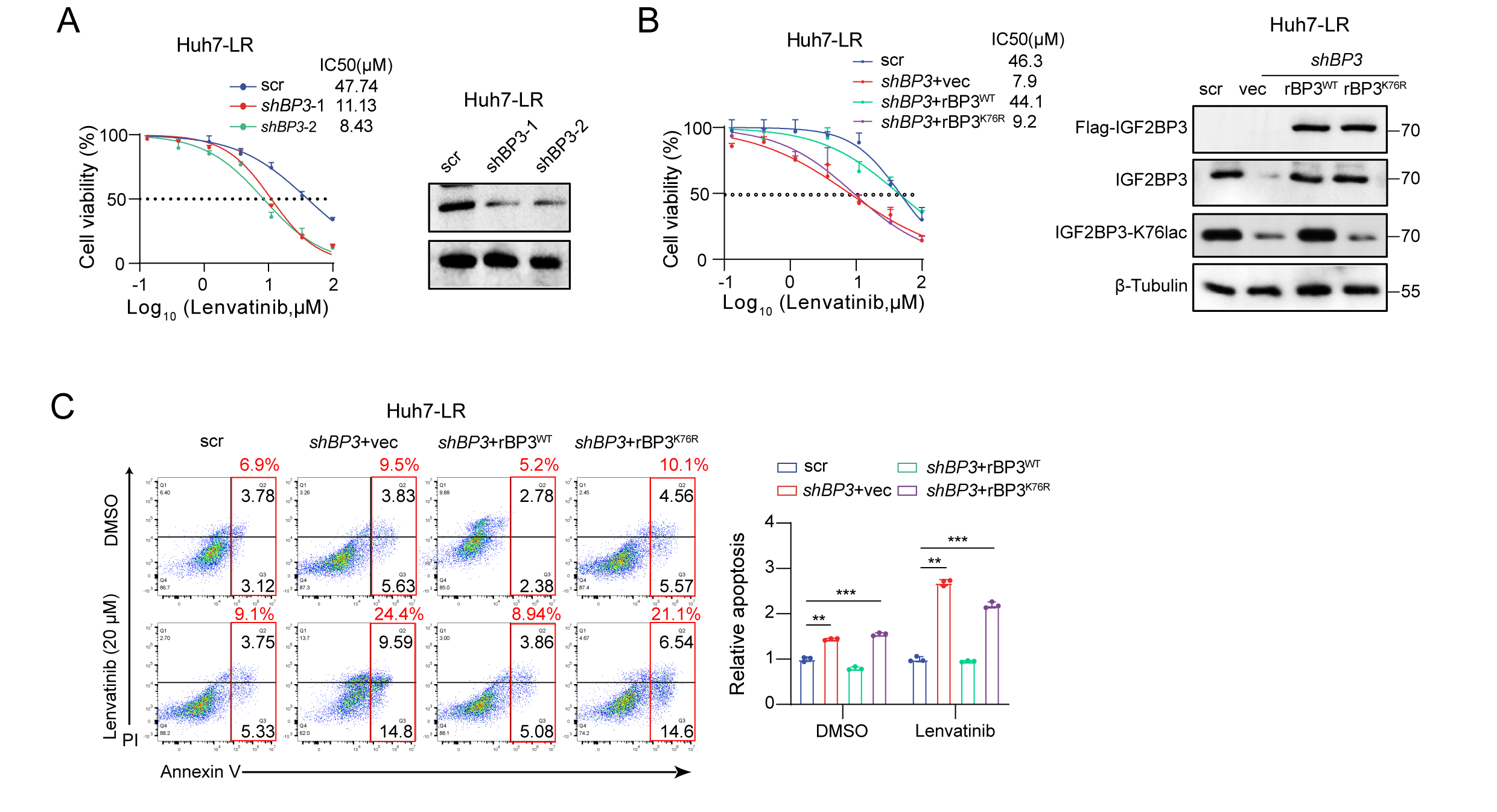
**Figure S2**

**Figure S3** IGF2BP3 lactylation modulates redox homeostasis in lenvatinib-resistant HCC via the upregulation of PCK2.

(A) Apoptotic response in Hep3B-LR cells after siRNA treatment, as assessed by flow cytometry via Annexin V/PI staining under lenvatinib (40 μM) treatment.

(B) Determination of the IC50 value in Huh7-LR cells using the CCK-8 assay following siRNA screening.

(C) Flow cytometry analysis of apoptosis in Huh7-LR cells after siRNA treatment with lenvatinib (40 μM) via Annexin V/PI staining.

(D) Western blot and qPCR analysis of PCK2 and NRF2 expression following IGF2BP3 inhibition in Huh7-LR cells. β-Actin and β-Tubulin served as loading controls.

(E) Impact of PCK2 inhibition on apoptosis (40 μM, lenvatinib) in Hep3B-LR-shBP3 (shIGF2BP3) cells expressing rBP3^WT^ (rIGF2BP3^WT^) analyzed via flow cytometry with Annexin V/PI staining.

(F) Effects of PCK2 inhibition on the IC50 and apoptosis value (40 μM, lenvatinib) in Huh7-LR-shBP3 cells transfected with rBP3^WT^.

(G) Comparison of GSH/GSSG levels and NADPH/NADP^+^ ratios in lenvatinib-resistant and parental cells.

(H and I) Western blot analysis of PCK2 expression (H) and ROS levels (I) in lenvatinib-resistant cells transfected with scr (scrambled) or shPCK2.

(J) GSH/GSSG and NADPH/NADP^+^ ratios in lenvatinib-resistant cells transfected with scr or shPCK2 constructs.

(K) ROS levels following PCK2 inhibition in Huh7-LR-shBP3 cells expressing rBP3^WT^.

Statistical significance was determined using two-tailed unpaired Student’s t-test or one-way analysis of variance (ANOVA). Each bar in the graphical representation indicates the mean ± SD. Statistical notation: ns (not significant), **P* < 0.05, ***P* < 0.01, ****P* < 0.001.

**
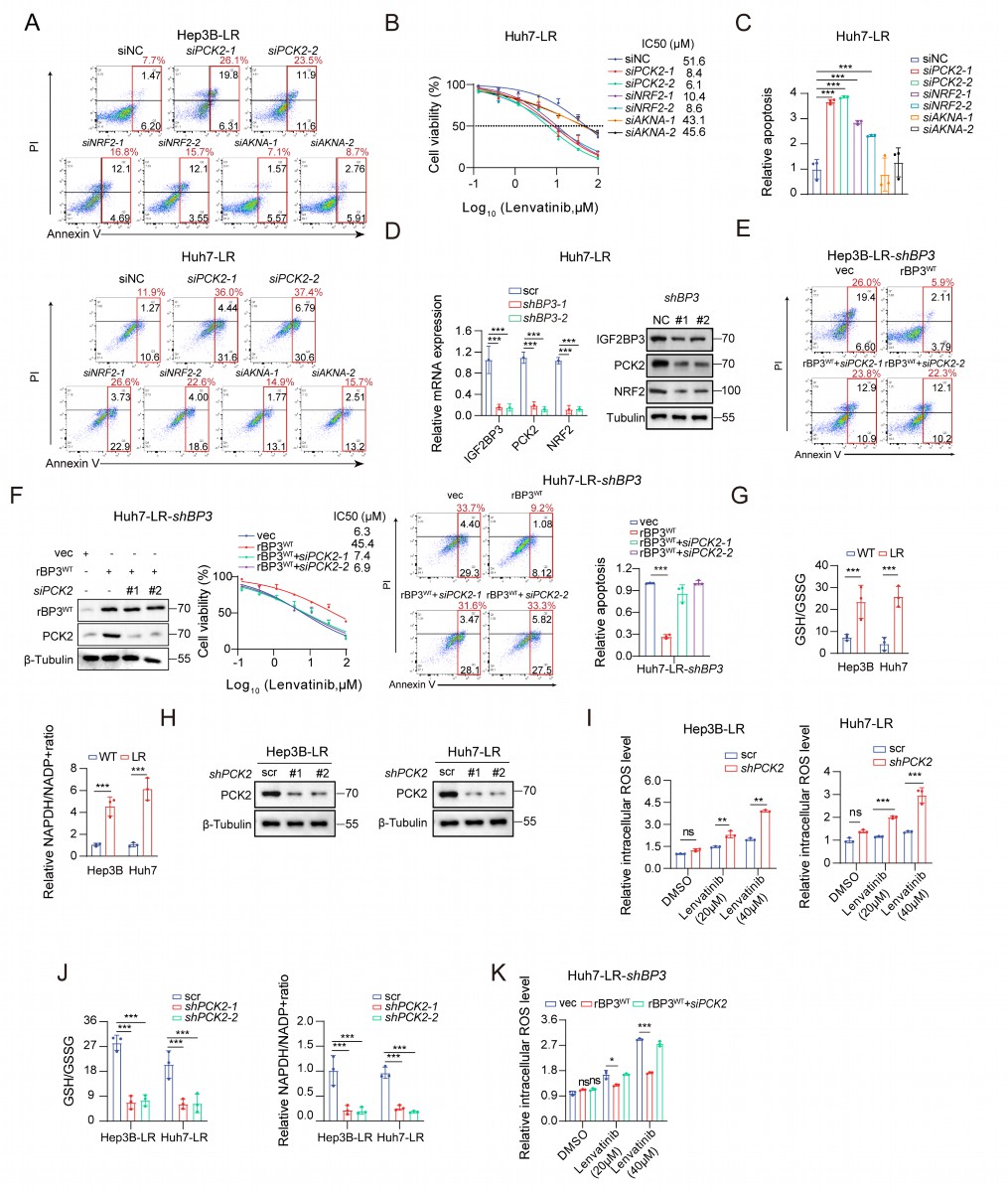
Figure S3**

**Figure S4** IGF2BP3 modulates PCK2 and NRF2 expression in a m6A-dependent manner.

(A and B) RIP‒qPCR evaluation of PCK2 and NRF2 mRNA enrichment with IGF2BP3 in Hep3B-LR and Huh7-LR (B) cells and the corresponding parental cells.

(C) Sequence-based RNA adenosine methylation site predictor (SRAMP) was used to predict m6A modification sites on the RNA sequences of PCK2 and NRF2.

(D) Mapping of IGF2BP3 binding sites and m6A motif predictions for PCK2 and NRF2 transcripts in Hep3B-LR cells.

(E) MeRIP‒qPCR analysis of m6A motif-specific enrichment in PCK2 and NRF2 mRNAs in Hep3B and Huh7 cells.

(F) Validation of m6A modification sites in PCK2 (P3 and P4) and NRF2 (N1 and N2) using stepwise mutations in luciferase reporter assays in Hep3B-LR cells.

(G) In vitro RNA pull-down assays demonstrating that IGF2BP3 binds to biotinylated PCK2 and NRF2 probes in Hep3B-LR and Huh7-LR cells, with β-Tubulin as a loading control.

(H) qPCR analysis of m6A-related writers, erasers, and readers in parental and lenvatinib-resistant cells.

(I) Assessment of PCK2 and NRF2 expression via qPCR and Western blotting following the inhibition of METTL3, METTL14, or WTAP in lenvatinib-resistant cells, with β-Tubulin used as a loading control.

Statistical significance was determined using two-tailed unpaired Student’s t-test or one-way analysis of variance (ANOVA). Each bar in the graphical representation indicates the mean ± SD (n = 3). Statistical notation: ns (not significant), **P* < 0.05, ***P* < 0.01, ****P* < 0.001.


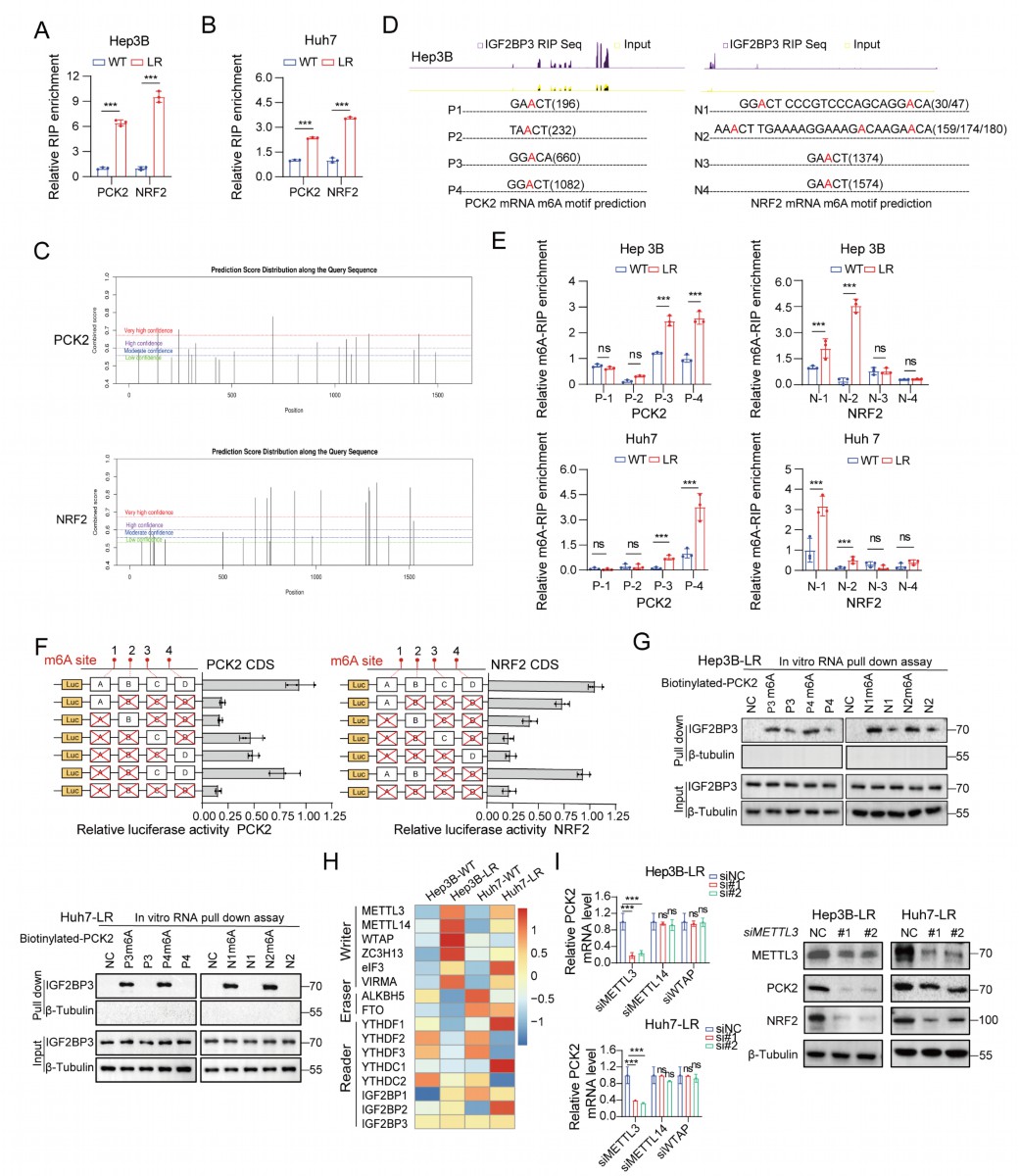
**Figure S4**

**Figure S5** IGF2BP3 mediates PCK2 and NRF2 upregulation in lenvatinib-resistant cell

(A) RIP-qPCR analysis using an IGF2BP3 antibody showing the enrichment of PCK2 and NRF2 mRNAs following METTL3 inhibition in Hep3B/Huh7-LR cells and the corresponding normal control (NC) cells.

(B and C) MeRIP-qPCR analysis demonstrating the enrichment of m6A motifs in PCK2 and NRF2 mRNAs following METTL3 inhibition in Hep3B-LR cells (B) and Huh7-LR cells (C).

(D-G) Degradation Rates of PCK2 and NRF2 mRNAs post-Actinomycin D exposure. Evaluation of PCK2 and NRF2 mRNA levels in IGF2BP3 knockdown (D, E) or METTL3 knockdown (F, G) in lenvatinib-resistant cells following actinomycin D treatment.

(H) Polysome fractionation was followed by qPCR assays using β-actin mRNA as a reference.

Statistical significance was determined using two-tailed unpaired Student’s t-test. Each bar in the graphical representation indicates mean ± SD (n = 3). Statistical notation: ns (not significant), **P* < 0.05, ***P* < 0.01, ****P* < 0.001.

**Figure S5**


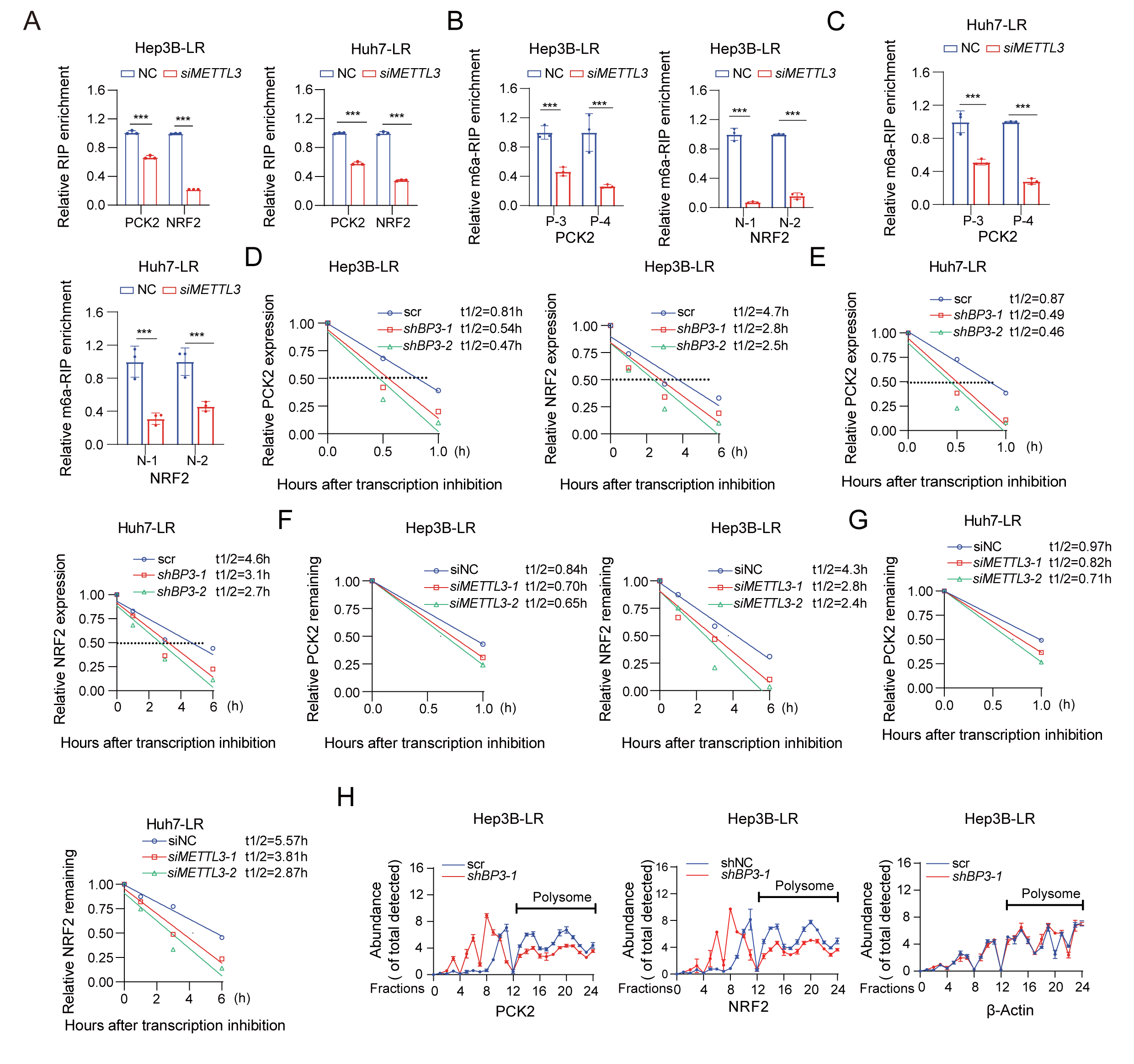


**Figure S6** Serine metabolism activation contributes to lenvatinib resistance

(A) GSEA depicting gene sets associated with glycine, serine, and threonine metabolism in the Huh7-LR cell line sourced from the GEO database (GSE186191). The normalized enrichment score (NES) is provided.

(B) Metabolite analysis via LC‒MS in Hep3B-LR-shBP3 (IGF2BP3) cells expressing rBP3^WT^ (rIGF2BP3^WT^) (n=3) or rBP3^K76R^ (rIGF2BP3^K76R^) (n=3). Dimensionality reduction analysis represents data with ellipse lines and center diamonds, indicating 95% confidence intervals (CIs) and means.

(C) LC‒MS analysis of intracellular metabolites in lenvatinib-resistant cell lines and paired parental cells.

(D and E) Detection of IC50 values (D) and apoptosis rates (E) in lenvatinib-resistant cells cultivated in serine-free medium.

(F) Measurement of ROS levels in lenvatinib-resistant cells cultivated in serine-free medium.

(G) Assessment of GSH/GSSG levels and NADPH/NADP^+^ ratios in lenvatinib-resistant cells grown in serine-free medium.

(H) qPCR analysis demonstrating elevated levels of key enzymes involved in de novo serine synthesis, cysteine and methionine metabolism, the glutathione synthesis pathway, and classic antioxidative stress-related pathways in lenvatinib-resistant cells.

(I) Measurement of ROS levels in lenvatinib-resistant cells transfected with shBP3.

(J and K) Measurement of ROS levels (J), the ratio of GSH/GSSG, and the NADPH/NADP+ ratio (K) in lenvatinib-resistant cells with shBP3 coexpressed with vec, rBP3^WT^, or rBP3^K76R^.

(L) IC50 values for Hep3B-LR-shBP3 cells transfected with rBP3^WT^ and treated with BSO (buthionine sulfoximine) (200 μM) or transfected with rBP3^K76R^ and treated with NAC (N-acetyl-L-cysteine) (5 mM).

(M) Western blot analysis of IGF2BP3 lactylation levels in subcutaneous tumors derived from Huh7-LR-shBP3+rBP3^WT^ or Huh7-LR-shBP3^K76R^ cells treated with PBS, serine (400 μM), or buthionine sulfoximine (BSO, an ROS scavenging inhibitor, 450 mg/kg) in conjunction with daily lenvatinib (10 mg/kg).

Statistical analysis was performed using two-tailed unpaired Student’s t-test or one-way analysis of variance (ANOVA). Each bar in the graph represents the mean ± SD (n = 3). Statistical notation: ns (not significant), **P* < 0.05, ***P* < 0.01, ****P* < 0.001.


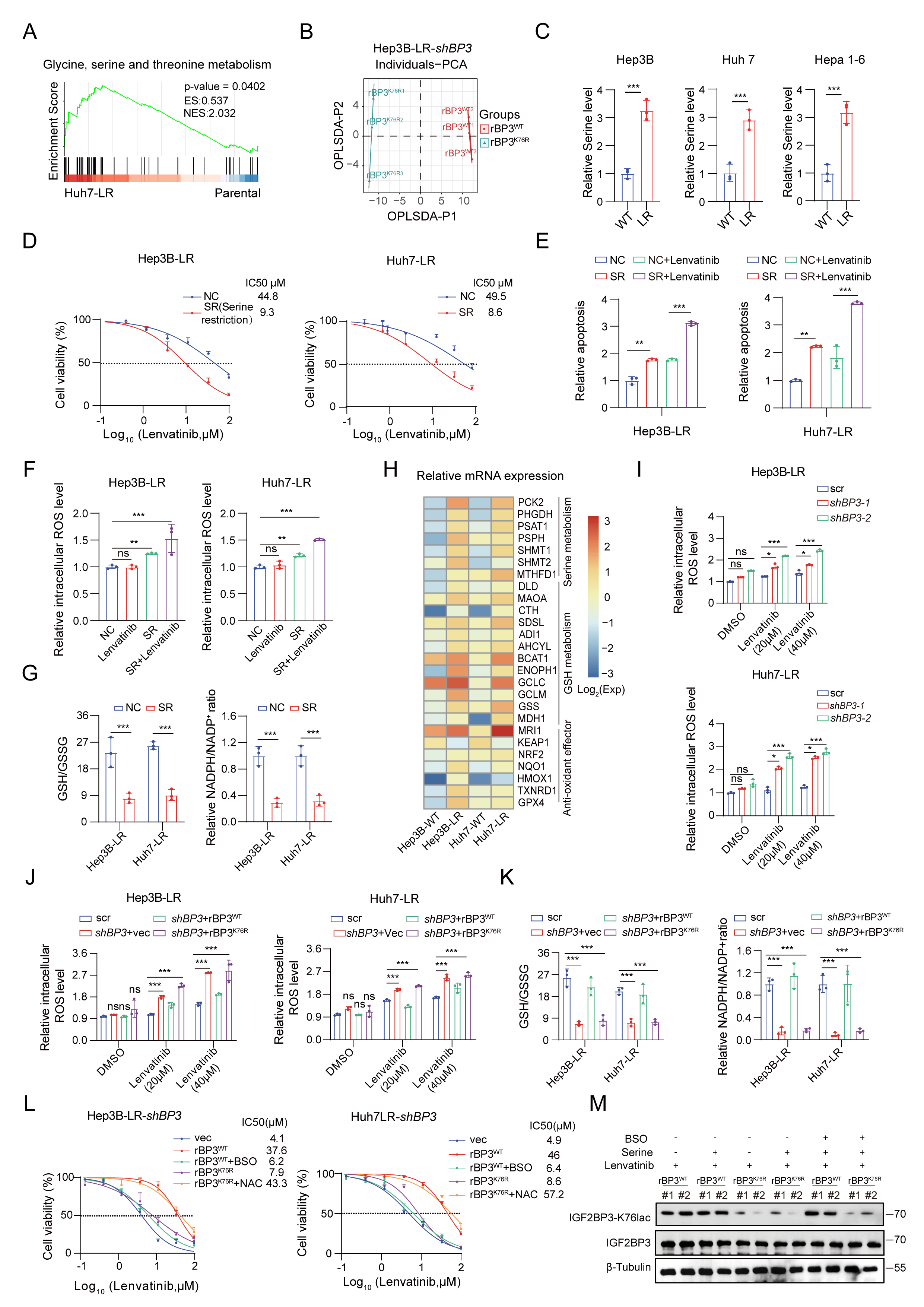
**Figure S6**

**Figure S7** IGF2BP3 lactylation fine-tunes serine metabolism through increasing the expression of PCK2

(A-C) Assessment of IC50 values (A) and apoptosis rates (B and C) in Hep3B-LR-shBP3 (IGF2BP3) or Huh7-LR-shBP3 cells transfected with vec, rBP3^WT^ (rIGF2BP3^WT^), rBP3^WT^ treated with NCT-503 (20 μM), rBP3^K76R^ (rIGF2BP3^K76R^), or rBP3^K76R^ treated with serine (400 μM).

(D) qPCR analysis demonstrating decreased levels of key enzyme mRNAs involved in de novo serine synthesis, the GSH synthesis pathway, and classic antioxidative stress-related pathways in the IGF2BP3-knockdown lenvatinib-resistant cells.

(E) Determination of IC50 values in Huh7-LR and Hep3B-LR cells transfected with siPCK2 or siPHGDH using the CCK-8 assay.

(F) Measurement of the GSH/GSSG and NADPH/NADP^+^ ratios in Hep3B-LR and Huh7-LR cells transfected with siPCK2 or siPHGDH.

(G) LC‒MS analysis of intracellular serine levels in Hep3B-LR and Huh7-LR cells transfected with siPCK2 or siPHGDH.

(H) RNA sequencing was used to compare the genetic profiles of scr (scrambled) and shPCK2 cells derived from Hep3B-LR. Gene set enrichment analysis (GSEA) revealed strong correlations between PCK2 and the glycine, serine, and threonine metabolism pathways.

(I) LC‒MS analysis of intracellular serine levels in PCK2-knockdown (KD) and scr lenvatinib-resistant cells (n = 3 biologically independent samples).

Statistical analysis was conducted using one-way analysis of variance (ANOVA). Each bar in the graph represents the mean ± SD (n = 3). Statistical notations: **P* < 0.05, ***P* < 0.01, ****P* < 0.001.

**
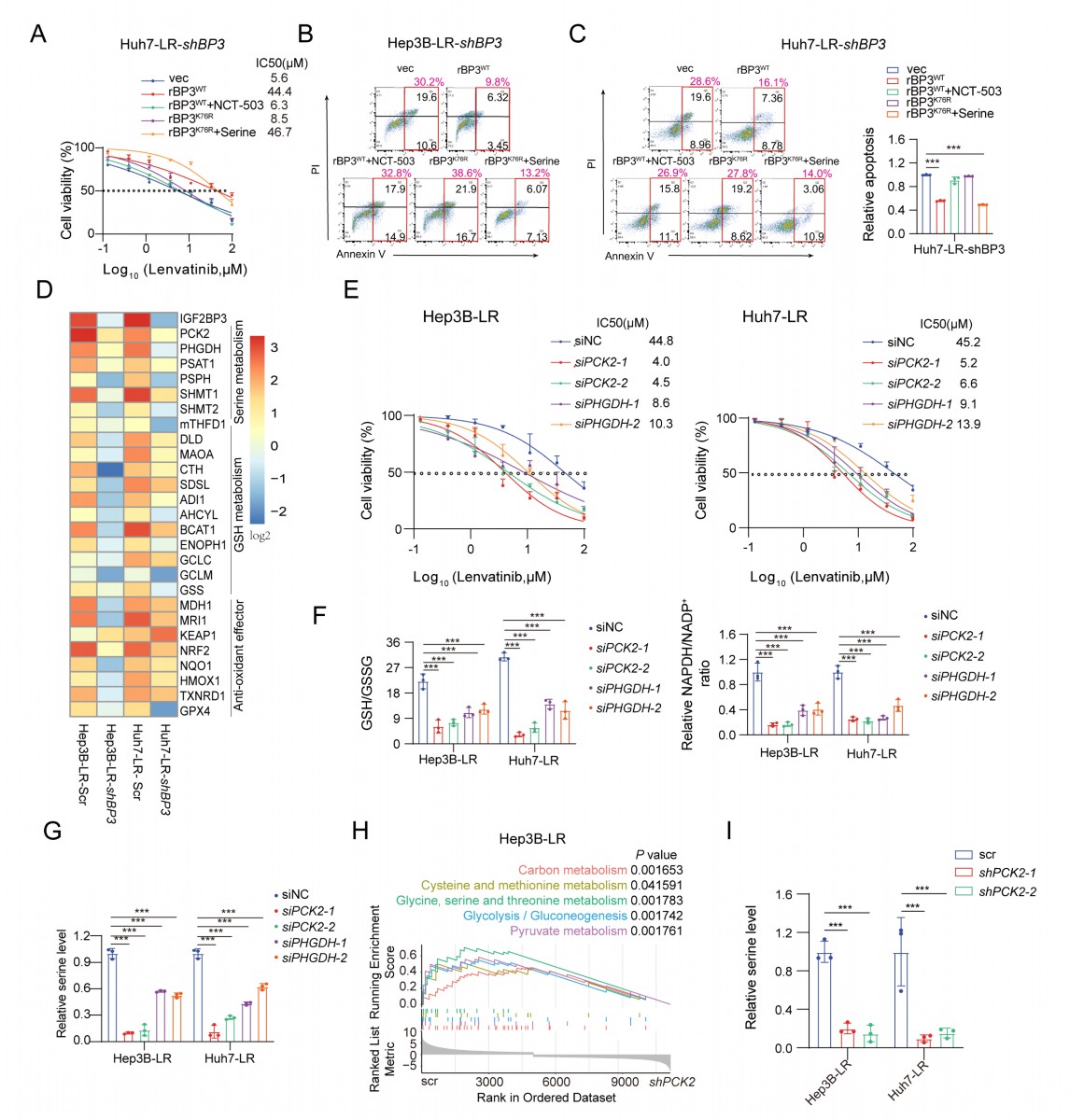
Figure S7**

**Figure S8** Lenvatinib-resistant cell lines exhibit increased SAM production and RNA m6A modification

(A) Detection of the intracellular SAM levels in lenvatinib-resistant and paired parental cells via ELISA (n = 3 biologically independent samples).

(B) KEGG analysis revealed a robust correlation between PCK2 and the S-adenosylmethionine metabolic process and mRNA methylation in RNA-seq data from scr (scrambled) and shPCK2 cells in Hep3B-LR.

(C) Measurement of the intracellular SAM level in lenvatinib-resistant cells transfected with shPCK2 using ELISA (n = 3 biologically independent samples).

(D) Immunoblotting analysis of the histone methylation markers H3K4me3, H3K9me3, and H3K27me3 in parental and lenvatinib-resistant cells, with histone H3 as a loading control. DNA dot blot assays were used to detect 5-methylcytosine (5-mC) in parental and lenvatinib-resistant cells.

(E) RNA m6A dot blot assays for parental and lenvatinib-resistant cells, with methylene blue (MB) staining as a loading control.

(F) Immunofluorescence imaging of m6A modifications in lenvatinib-resistant (PD) or lenvatinib-sensitive (PR) patients.

(G) Western blot analysis of the histone methylation markers H3K4me3, H3K9me3, and H3K27me3 in Hep3B/Huh7-LR-shBP3 (IGF2BP3) cells expressing rBP3^WT^ (rIGF2BP3^WT^) or rBP3^K76R^ (rIGF2BP3^K76R^), with histone H3 as a loading control. Or DNA dot blot assays for 5-methylcytosine (5-mC) in Hep3B/Huh7-LR-shBP3 cells expressing rBP3^WT^ or rBP3^K76R^.

(H) IC50 values for lenvatinib-resistant cells treated with the m6A inhibitor DAA (3-deazaadenosine), the DNA methylation inhibitor 5-azacytidine, or the histone methylation inhibitor DZNep (3-deazaneplanocin A).

(I) RNA m6A dot blot assays in lenvatinib-resistant cells transfected with shPCK2.

Statistical analysis was performed using two-tailed unpaired Student’s t-test or one-way analysis of variance (ANOVA). Each bar in the graph represents the mean ± SD (n = 3). Statistical notations: **P* < 0.05, ***P* < 0.01, ****P* < 0.001.

**
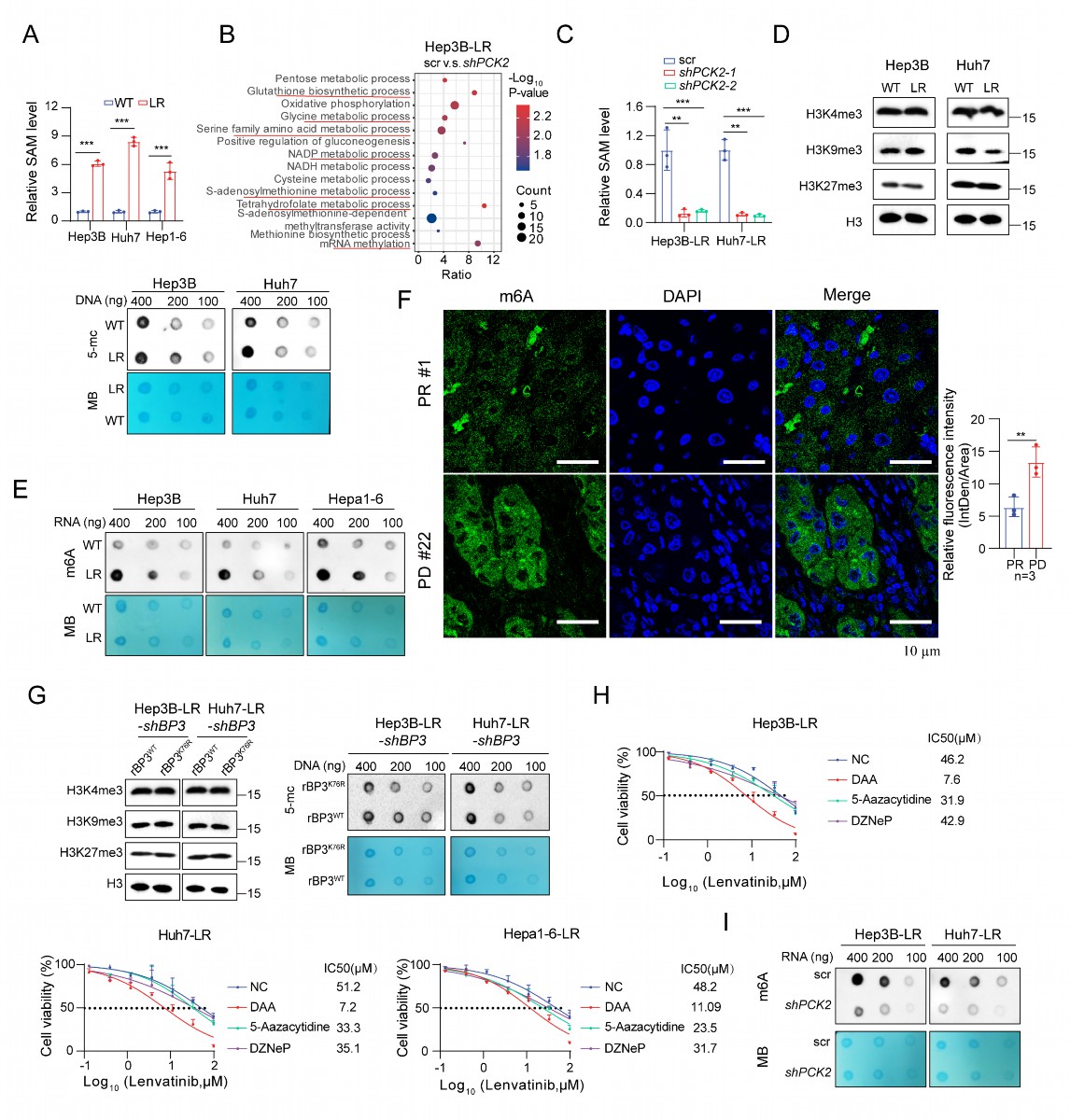
Figure S8**

**Figure S9** Lactylated IGF2BP3 enhances gluconeogenesis in lenvatinib-resistant cell lines

(A) Quantification of 2-NBDG uptake in Hep3B/Huh7-LR-shBP3 (IGF2BP3) cells transfected with rBP3^WT^ (rIGF2BP3^WT^) or rBP3^K76R^ (rIGF2BP3^K76R^).

(B) Evaluation of lactate production in Hep3B/Huh7-LR-shBP3 cells transfected with rBP3^WT^ or rBP3^K76R^.

(C) Analysis of 2-NBDG uptake in lenvatinib-resistant cells transfected with shPCK2.

(D) Measurement of lactate production in lenvatinib-resistant cells transfected with shPCK2.

(E) Incubation of cells with ^13^C-labeled glucose for 24 hours, followed by the analysis of metabolite labeling via liquid chromatography‒mass spectrometry (LC‒MS).

Statistical analysis was performed using two-tailed unpaired Student’s t-test. Each bar in the graph represents the mean ± SD (n = 3). Statistical notation: n (not significant).


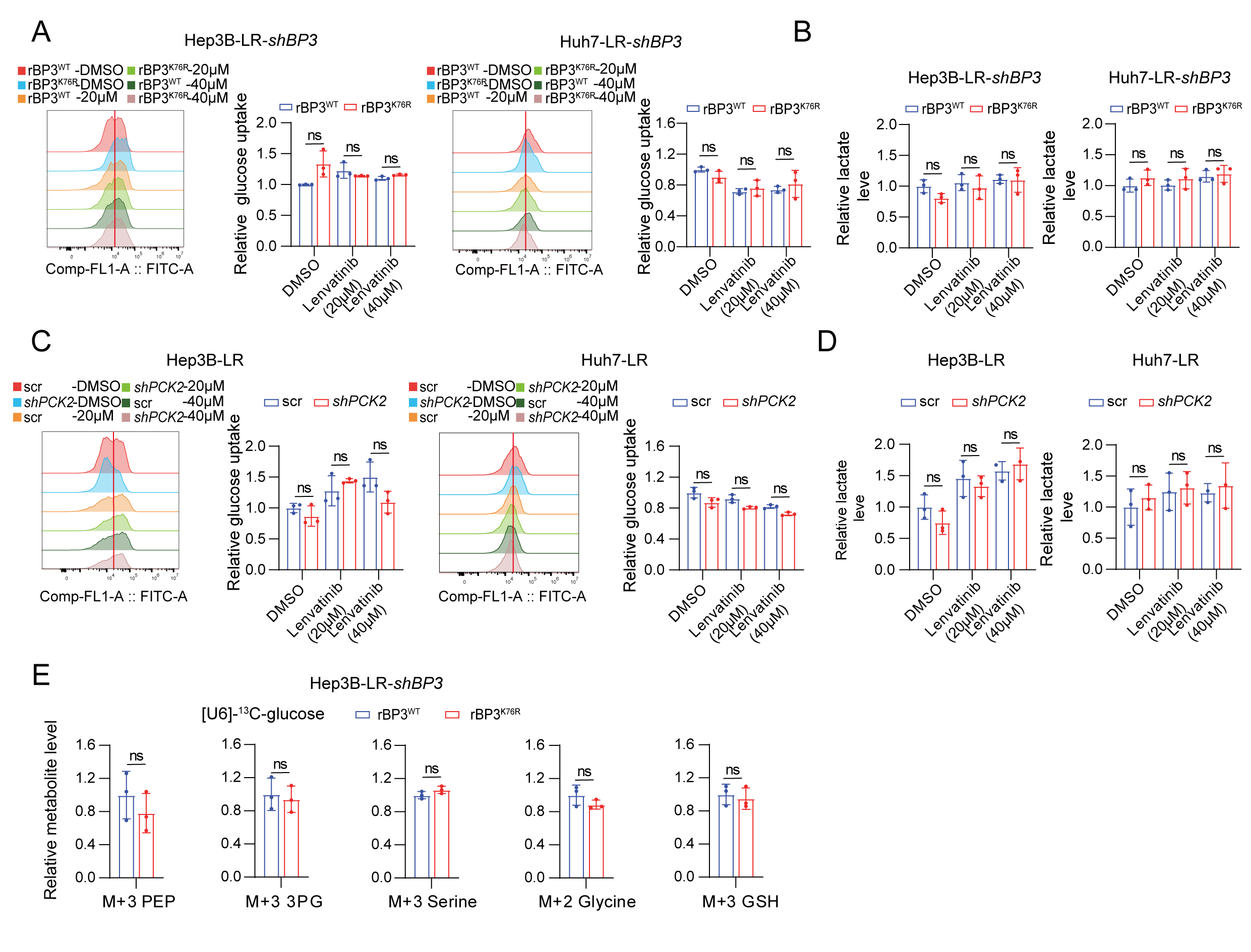
**Figure S9**

**Figure S10** PCK2 and lactylated IGF2BP3 facilitate NRF2 expression via SAM-dependent m6A modification

(A) RIP enrichment of PCK2 and NRF2 mRNAs after serine restriction (SR) in Huh7-LR cells.

(B) MeRIP‒qPCR analysis of specific m6A motif enrichment in PCK2 and NRF2 mRNAs after serine restriction in Huh7-LR cells.

(C) MeRIP‒qPCR analysis of specific m6A motif enrichment of PCK2 and NRF2 mRNAs after PCK2 OE in Huh7-WT cells.

(D) RIP analysis revealed enrichment of PCK2 and NRF2 mRNAs in Huh7-WT cells transfected with PCK2 OE following inhibition of METTL3 or IGF2BP3 (siBP3) or treatment with 3-deazaadenosine (DAA) (50 μM).

(E) RNA m6A dot blot assays and Western blot analysis of PCK2 and NRF2 in Huh7-WT cells transfected with PCK2 OE following IGF2BP3 or METTL3 inhibition or treated with PBS, DAA (50 μM) or NCT-503 (20 μM). Methylene blue (MB) staining and β-Tubulin served as loading controls.

(F) MeRIP‒qPCR analysis of m6A motif enrichment in PCK2 and NRF2 mRNAs after PCK2 inhibition in Huh7-LR cells.

(G) RIP analysis of PCK2 and NRF2 mRNAs in Huh7-LR cells with PCK2 inhibition rescued with rBP3^WT^ (rIGF2BP3^WT^), rBP3^K76R^ (rIGF2BP3^K76R^) or serine.

(H) RNA m6A dot blot assays and Western blot analysis of PCK2 and NRF2 in Huh7-LR cells with PCK2 inhibition treated with 3PG (0.75 mM), serine (400 μM), methionine (100 μM), or SAM (50 μM). Methylene blue staining and β-Tubulin served as loading controls.

(I) RNA m6A dot blot assays and Western blot analysis of PCK2 and NRF2 in Huh7-LR cells with PCK2 inhibition and those transfected with rBP3^WT^ or rBP3^K76R^. Methylene blue staining and β-Tubulin were used as loading controls.

(J) Western blot analysis of PCK2 levels in Hep3B-LR and Huh7-LR cells transfected with siNRF2.

(K) Correlation analysis between PCK2 and NRF2 mRNA expression in the TCGA-LIHC dataset (n=369).

(L) Correlation analysis between PCK2 and NRF2 mRNA expression in HCC cell lines in the Liver Cancer Model Repository (LIMORE) database.

Statistical significance was assessed using two-tailed unpaired Student’s t-test or one-way analysis of variance (ANOVA). Spearman's rank correlation test was used for correlation analysis. Each bar in the graph represents the mean ± SD (n = 3). Statistical notation: ns (not significant), **P* < 0.05, ***P* < 0.01, ****P* < 0.001.


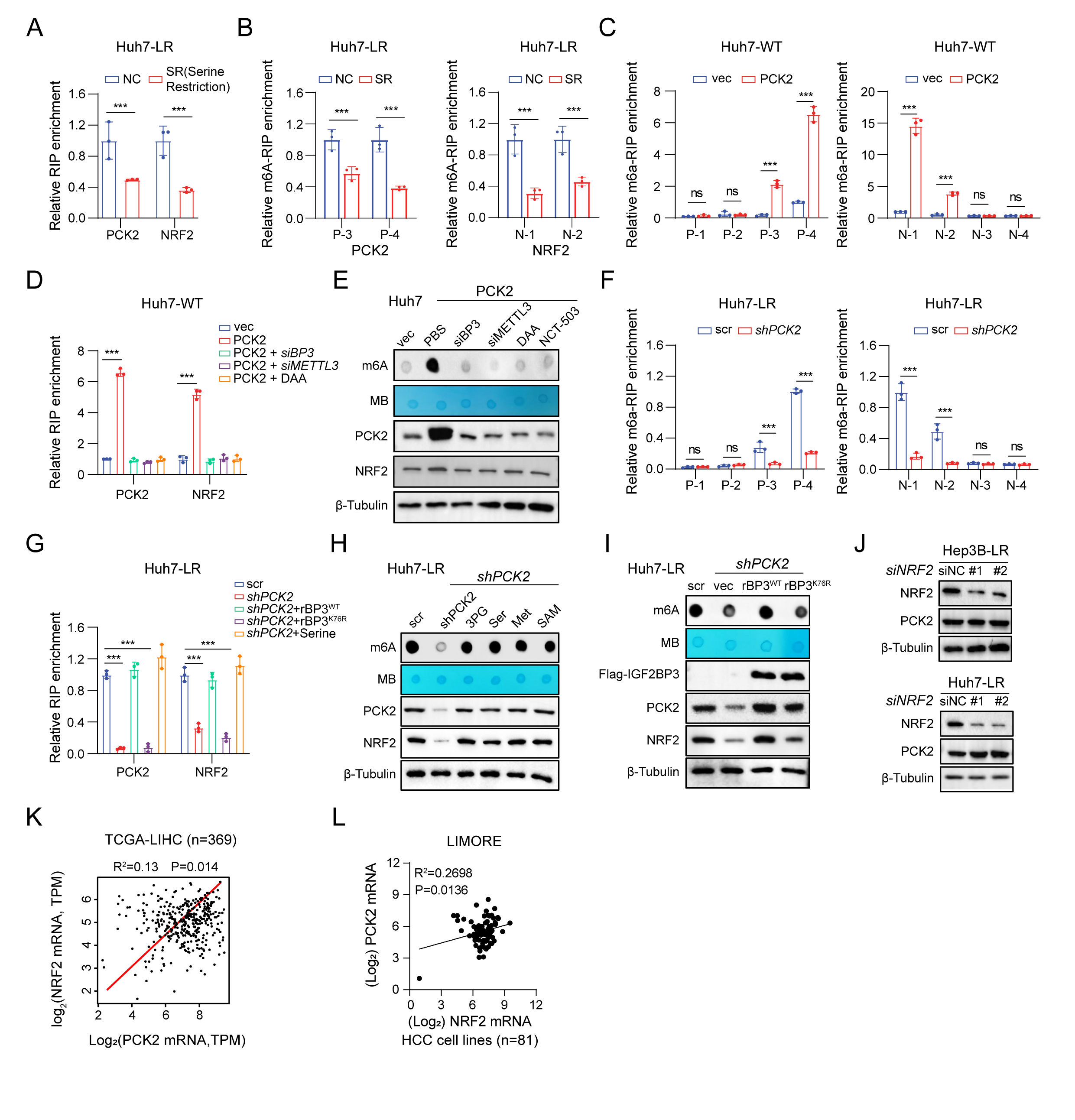
**Figure S10**

**Figure 8** High IGF2BP3 lactylation levels correlate with poor therapeutic responses to lenvatinib, and targeting IGF2BP3 restores the lenvatinib response in HCC.

(A) CT images of HCC patients demonstrating partial response (PR) and progressive disease (PD) in response to lenvatinib treatment. Scale bar: 15 cm.

(B) Immunofluorescence staining of representative lenvatinib-sensitive (PR+SD, n=15) and lenvatinib-resistant (PD, n=22) patient samples. Confocal microscopy images showing colocalization of IGF2BP3 (red) and pan-Klac (green) with nuclear staining by DAPI (blue) (scale bar, 10 µm). For colocalization analysis, ImageJ was used, and the mean fluorescence intensity (MFI) of Klac staining of IGF2BP3 was statistically analyzed via two-tailed paired t test.

(C) AUC curves for predicting the response to lenvatinib via the mean fluorescence intensity (MFI) of Klac staining of IGF2BP3 (IGF2BP3lac) in posttreatment tumor samples with modified RECIST criteria.

(D and E) Evaluation of lenvatinib response in the Tongji Hospital cohort based on modified RECIST criteria (D). Waterfall plots displaying intrahepatic lesion responses in subgroups with high (n = 24) and low (n = 13) IGF2BP3lac levels treated with lenvatinib (E).

(F and G) Kaplan‒Meier survival curves depicting progression-free survival (PFS) (F) and overall survival (OS) (G) relative to IGF2BP3lac expression were generated using SPSS 25.0.

(H) Representative images of the Huh7-LR cell-derived orthotopic HCC model at the endpoint (n=5 per group). The mice were intraperitoneally injected with 100 mg/kg 2-DG every day for two weeks in combination with the oral administration of lenvatinib at 10 mg/kg.

(I) Western blot analysis of IGF2BP3 lactylation levels in the Huh7-LR cell-derived orthotopic HCC model treated with 2-DG alone or in combination with lenvatinib.

Statistical significance was assessed via two-tailed unpaired Student’s t test or one-way analysis of variance (ANOVA). Each bar represents the mean ± SD. Statistical notations: **P* < 0.05, ***P* < 0.01, ****P* < 0.001.

**Figure 8**

**
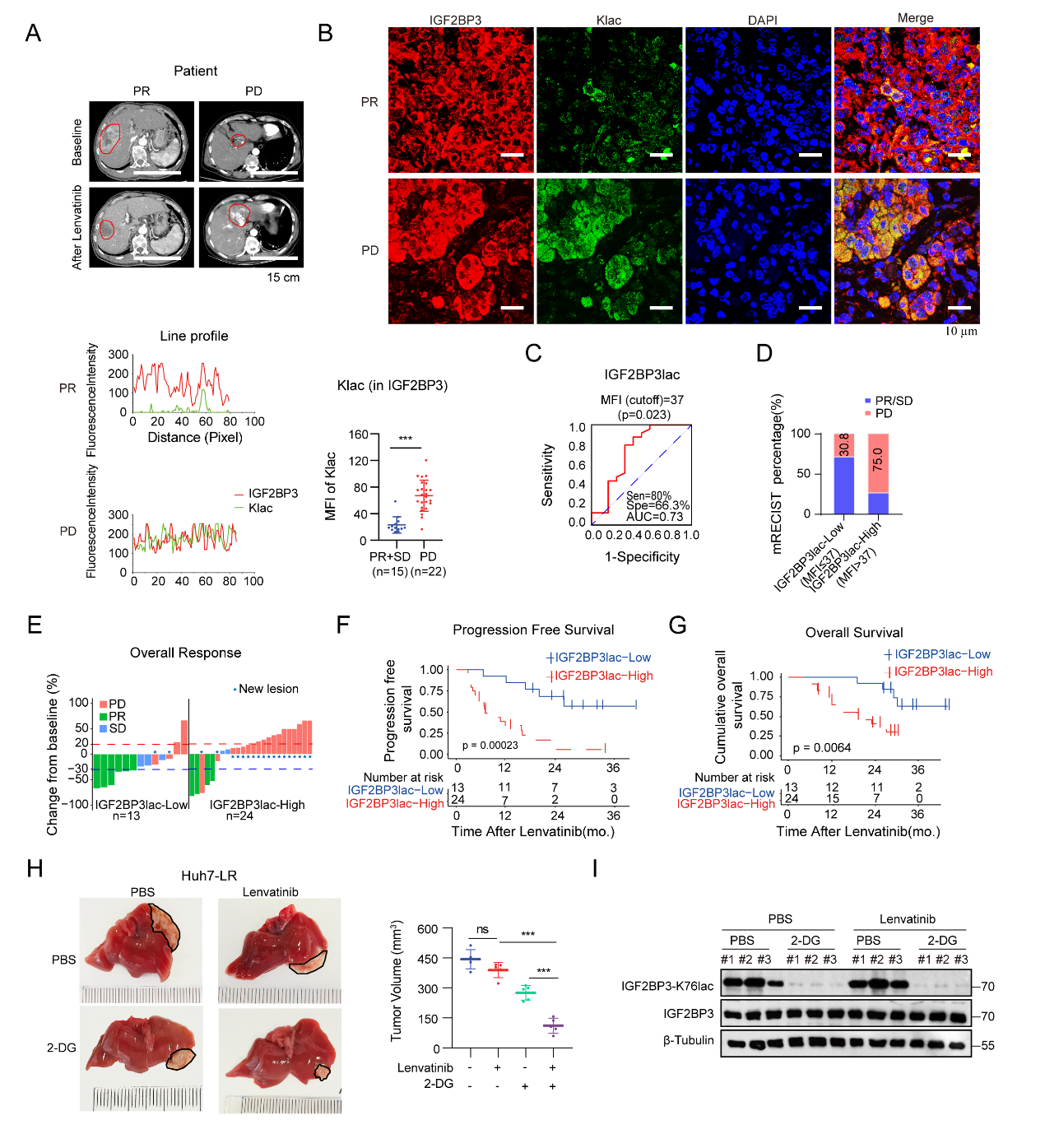
**

**Figure S11**

(A) Schematic representation of LNPs (lipid nanoparticles) carrying IGF2BP3 siRNA.

(B) Description of nanoparticle preparation, characterized by an entrapment efficiency exceeding 90% for siRNA loading and a zeta potential of 4.3 mV.

(C and D) Distribution graph showing the normal range of hydrodynamic diameters of the nanoparticles (C). Transmission electron microscopy (TEM) image illustrating the morphology of the prepared nanoparticles (D).

(E) Quantitative analysis of alanine aminotransferase (ALT) and aspartate aminotransferase (AST) levels in BALB/C nude mice treated with siNC- or siIGF2BP3-loaded liposomes.

(F) Temporal changes in IGF2BP3 and PCK2 expression were assessed (at 0, 1, 3, and 6 days) in orthotopic liver tumors (BALB/c nude mice) treated with IGF2BP3 siRNA-loaded liposomes.

(G) Biodistribution of liposomes in orthotopic HCC model mice via tail vein injection monitored via an in vivo imaging system (IVIS) at different time points (0, 1, 3, and 6 days). Representative images of the Huh7-LR cell-derived orthotopic HCC model at the endpoint (n=5 per group). The mice were treated with siIGF2BP3-loaded liposomes every 3 days for two weeks in combination with the oral administration of lenvatinib at 10 mg/kg.

Statistical significance was assessed using one-way analysis of variance (ANOVA). Each bar represents the mean ± SD. Statistical notations: **P* < 0.05, ***P* < 0.01, ****P* < 0.001.


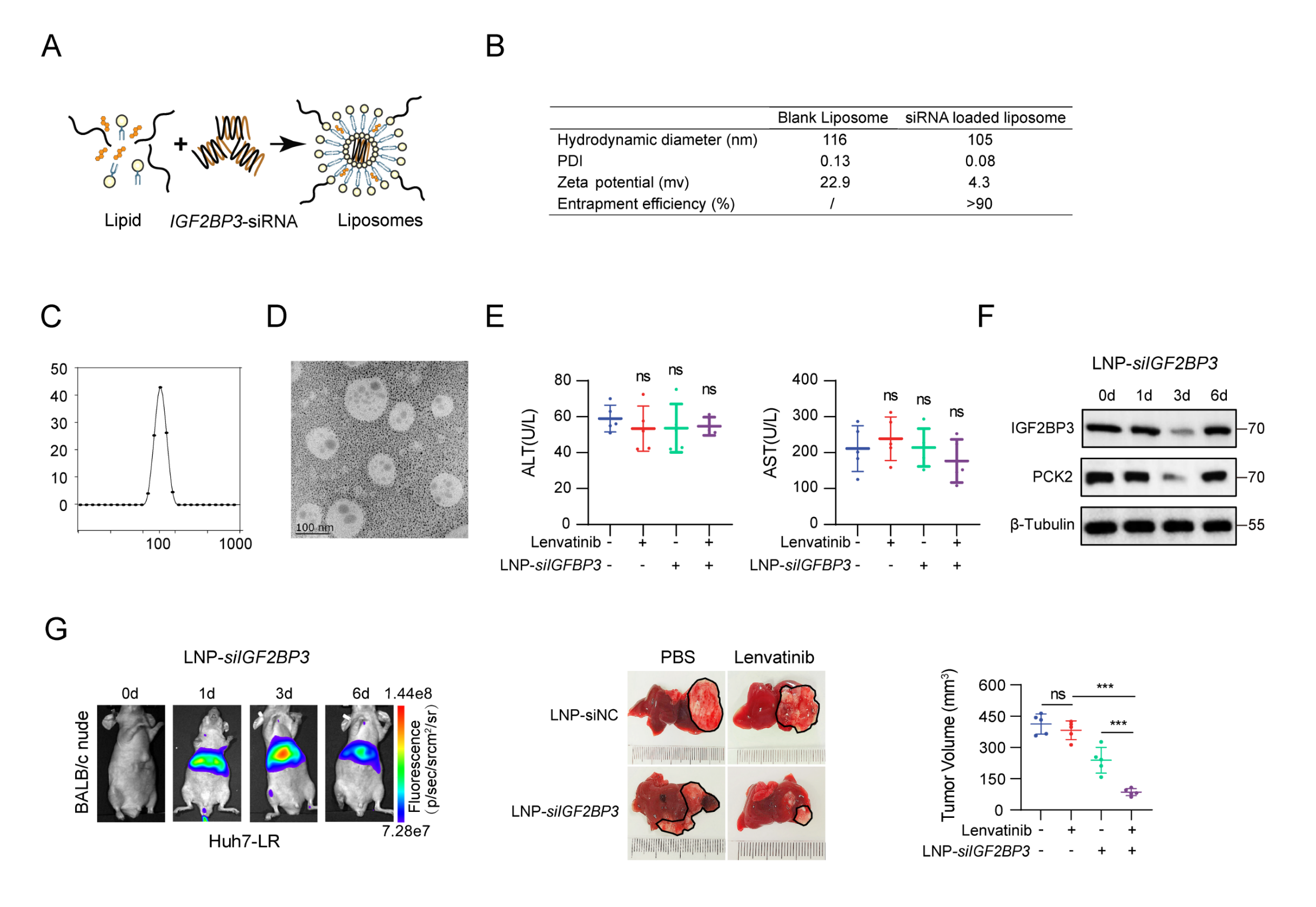
**Figure S11**

**Table S1. Sequences of siRNAs/shRNA.**

| PCK2 human #1 | 5'-GTAGAGAGCAAGACGGTGATT-3' |
| --- | --- |
| PCK2 human #2 | 5'-CCTCGTATCTTCCATGTCAAT-3' |
| NRF2 human #1 | 5'-CCGGCATTTCACTAAACACAA-3' |
| NRF2 human #2 | 5'-CCCGAATTACAGTGTCTTAAT-3' |
| AKNA human #1 | 5'-GCTCAGAAACAAGCAGAGTTT-3' |
| AKNA human #2 | 5'-CCAATACACAGGCCACGAATA-3' |
| METTL3 human #1 | 5'- GCACTTGGATCTACGGAAT -3' |
| METTL3 human #2 | 5'- CGACTACAGTAGCTGCCTT -3' |
| METTL14 human #1 | 5'- CAACTACAATGCAGAAACA -3' |
| METTL14 human #2 | 5'- GAAGACGCCTTCATCTATT -3' |
| WTAP human #1 | 5'-GTTATGGCAAGAGATGAGTTA-3' |
| WTAP human #2 | 5'-ATGGCAAGAGATGAGTTAATT-3' |
| PHGDH human #1 | 5'-UAGCAAAGAGGAGCUGAUATT -3' |
| PHGDH human #2 | 5'-GACUUCACUGGUGUCAGAUTT-3' |
| IGF2BP3 human #1 | 5'-GCTGAGAAGTCGATTACTA-3' |
| IGF2BP3 human #2 | 5'-TAAGGAAGCTCAAGATATA-3' |

**Table** **S2. Primer sequences of HCC lenvatinib resistance-related gene.**

| Primer name | Sense (5’→3’) | Anti-sense (5’→3’) |
| --- | --- | --- |
| GCLM | TGTCTTGGAATGCACTGTATCTC | CCCAGTAAGGCTGTAAATGCTC |
| ENOPH1 | TGTCCCTGGATCGAAAGACCA | CCTGACTGCTGGAACTACATCT |
| AHCYL1 | TCGCTCGATCTCACAGTCCT | TCCCGGCGTCCAAATTCTG |
| NRF2 | TCAGCGACGGAAAGAGTATGA | CCACTGGTTTCTGACTGGATGT |
| MDH1 | GGTGCAGCCTTAGATAAATACGC | AGTCAAGCAACTGAAGTTCTCC |
| GSS | GGGAGCCTCTTGCAGGATAAA | GAATGGGGCATAGCTCACCAC |
| GCLC | GGAGGAAACCAAGCGCCAT | CTTGACGGCGTGGTAGATGT |
| BCAT1 | GCTCAACATGGACCGGATG | CCGCACATAGAGGCTGGTG |
| mTHFD1 | GCGCCAGCAGAAATCCTGA | AGGTACTTGCTCCTTCAACTGA |
| PCK2 | GCCATCATGCCGTAGCATC | AGCCTCAGTTCCATCACAGAT |
| MRI1 | GATCCCCGCCACCCTTATC | GTCTCCAGACGGAGGTCACAT |
| GPX4 | GAGGCAAGACCGAAGTAAACTAC | CCGAACTGGTTACACGGGAA |
| PSPH | GAGGACGCGGTGTCAGAAAT | GGTTGCTCTGCTATGAGTCTCT |
| SDSL | GACGGCTGGGAGAATGTCC | ATGGCCGCATTGAAGCAGT |
| MAOA | GAATCAAGAGAAGGCGAGTATCG | GGCAGCAGATAGTCCTGAAATG |
| NQO1 | GAAGAGCACTGATCGTACTGGC | GGATACTGAAAGTTCGCAGGG |
| SHMT1 | CTGGCACAACCCCTCAAAGA | AGGCAATCAGCTCCAATCCAA |
| KEAP1 | CTGGAGGATCATACCAAGCAGG | GGATACCCTCAATGGACACCAC |
| PHGDH | CTGCGGAAAGTGCTCATCAGT | TGGCAGAGCGAACAATAAGGC |
| ADI1 | CTCGGGGTGCTCTACTGGAA | TCCAAGTGCAAATGCTCCTCG |
| DLD | CTCATGGCCTACAGGGACTTT | GCATGTTCCACCAAGTGTTTCAT |
| SHMT2 | CCCTTCTGCAACCTCACGAC | TGAGCTTATAGGGCATAGACTCG |
| TXNRD1 | ATATGGCAAGAAGGTGATGGTCC | GGGCTTGTCCTAACAAAGCTG |
| HMOX1 | AAGACTGCGTTCCTGCTCAAC | AAAGCCCTACAGCAACTGTCG |
| CTH | AAAGACGCCTCCTCACAAGG | AAGGCAATTCCTAGTGGGATTTC |
| PSAT1 | AAAAACAATGGAGGTGCCGC | GGCTCCACTGGACAAACGTA |

**Table S3. Primer sequences of m6A-related gene.**

| Primer name | Sense (5’→3’) | Anti-sense (5’→3’) |
| --- | --- | --- |
| METTL3 | TTGTCTCCAACCTTCCGTAGT | CCAGATCAGAGAGGTGGTGTAG |
| METTL14 | AGTGCCGACAGCATTGGTG | GGAGCAGAGGTATCATAGGAAGC |
| WTAP | CTTCCCAAGAAGGTTCGATTGA | TCAGACTCTCTTAGGCCAGTTAC |
| ZC3H13 | TCTGATAGCACATCCCGAAGA | CAGCCAGTTACGGCACTGT |
| eIF3 | AGAGTAGAGCGCCTGTACCA | CAAGGCGACGCTGTTTTTCA |
| VIRMA | CGAGCGCTGAGCAAAGTTC | GGAGTTCTTGGTCCCCTTGG |
| ALKBH5 | CGGCGAAGGCTACACTTACG | CCACCAGCTTTTGGATCACCA |
| FTO | ACTTGGCTCCCTTATCTGACC | TGTGCAGTGTGAGAAAGGCTT |
| YTHDF1 | ACCTGTCCAGCTATTACCCG | TGGTGAGGTATGGAATCGGAG |
| YTHDF2 | AGCCCCACTTCCTACCAGATG | TGAGAACTGTTATTTCCCCATGC |
| YTHDF3 | TCAGAGTAACAGCTATCCACCA | GGTTGTCAGATATGGCATAGGCT |
| YTHDC2 | AGGACATTCGCATTGATGAGG | CTCTGGTCCCCGTATCGGA |
| IGF2BP1 | GCGGCCAGTTCTTGGTCAA | TTGGGCACCGAATGTTCAATC |
| YTHDC1 | AACTGGTTTCTAAGCCACTGAGC | GGAGGCACTACTTGATAGACGA |
| IGF2BP2 | AGTGGAATTGCATGGGAAAATCA | CAACGGCGGTTTCTGTGTC |
| IGF2BP3 | TATATCGGAAACCTCAGCGAGA | GGACCGAGTGCTCAACTTCT |
| HNRNPD | GCGTGGGTTCTGCTTTATTACC | TTGCTGATATTGTTCCTTCGACA |
| HNRNPC | TCCTCCTCCTATTGCTCGGG | GTGTTTCCTGATACACGCTGA |
| SRRM1 | AACAGGATAATCGGTTCAGCAAC | CCCAAGGATTTCCGTTACTCTTT |

**Table S4. MeRIP-qPCR primers for the IGF2BP3-targeted m6A site.**

| PCK2-P1-Forward | TCCCACTGGCATTCGAGATTT |
| --- | --- |
| PCK2-P1-Reverse | CAGTGTGGCAGTATTCTC |
| PCK2-P2-Forward | CAGCAGGGCCTCATCCGAAAG |
| PCK2-P2-Reverse | CCCAGCTGCCCACGGGCCCCAC |
| PCK2-P3-Forward | TCCCCGCTGTCCCGCATCGGG |
| PCK2-P3-Reverse | GAGTTGCCACCATAGCCGCTG |
| PCK2-P4-Forward | GTGCTAGACTGGATCTGCCGG |
| PCK2-P4-Reverse | TTGACCTGCTCTGTCAGGTAG |
| NRF2-N1-Forward | ATGATGGACTTGGAGCTGCCG |
| NRF2-N1-Reverse | CTCGACTTACTCCAAGATCTA |
| NRF2-N2-Forward | GACTTCAGTCAGCGACGGAAA |
| NRF2-N2-Reverse | GGAATGTGGGCAACCTGG |
| NRF2-N3-Forward | CAGAGCACTCACGTGCATGATG |
| NRF2-N3-Reverse | CTCTTTGGACATCATTTCGTT |
| NRF2-N4-Forward | AATGAAGCTCAACTTGCATTA |
| NRF2-N4-Reverse | AGTATTCACTAGGAGAATAA |

**Table S5. RNA probe sequence.**

| PCK2 | P3-m6A | 5'biotin-CCAGCCCCTGACAGGA(m6A)CAAGGGGAGCCA-3' |
| --- | --- | --- |
|  | P3-A | 5'biotin-CCAGCCCCTGACAGGACAAGGGGAGCCA-3' |
|  | P3-T | 5'biotin-CCAGCCCCTGACAGGTCAAGGGGAGCCA-3' |
|  | P4-m6A | 5'biotin-CTCCCTCCCCAAGGA(m6A)CTTCTGGGAACAGGA-3' |
|  | P4-A | 5'biotin-CTCCCTCCCCAAGGACTTCTGGGAACAGGA-3' |
|  | P4-T | 5'biotin- CTCCCTCCCCAAGGTCTTCTGGGAACAGGA-3' |
| NRF2 | N1-m6A | 5'biotinCCGCCGGGA(m6A)CTCCCGTCCCAGCAGGA(m6A)CATGGATTT-3' |
|  | N1-A | 5'biotin- CCGCCGGGACTCCCGTCCCAGCAGGACATGGATTT-3' |
|  | N1-T | 5'biotin- CCGCCGGGTCTCCCGTCCCAGCTGGACATGGATTT-3' |
|  | N2-m6A | 5'biotin-AAAACTTGAAAAGGAAAGA(m6A)CAAGAACAACTCCAA-3' |
|  | N2-A | 5'biotin- AAAACTTGAAAAGGAAAGACAAGAACAACTCCAA-3' |
|  | N2-T | 5'biotin- AAAACTTGAAAAGGAAAGTCAAGAACAACTCCAA -3' |

**Table S6. Antibodies used in this study.**

| Antibodies | Manufacturer | Application | Cat No. |
| --- | --- | --- | --- |
| Anti-L-Lactyl Lysine Rabbit mAb | Jingjie PTM BioLabs | 1:1000 for WB;  1:50 for IP;  1:100 for IF | Cat# PTM-1401RM;  RRID: AB_2942013 |
| Rabbit anti-human IGF2BP3 | Proteintech | 1:1000 for WB;  1:50 for RIP; | Cat# 14642-1-AP;  RRID: AB_2122782 |
| Mouse anti-human IGF2BP3 | HUABIO | 1:100 for IF | Cat# EM1701-15;  RRID: AB_3068743 |
| Mouse anti-Flag | Sigma-Aldrich, St. Louis | 1:1000 for WB;  1:50 for IP | Cat# F1804;  RRID: AB_262044 |
| Rabbit anti-human PCK2 | Proteintech | 1:1000 for WB | Cat# 14892-1-AP;RRID: AB_2160044 |
| Rabbit anti-human β-Tubulin | Proteintech | 1:1000 for WB | Cat# 10094-1-AP;RRID: AB_2210695 |
| Rabbit anti-human NRF2 | Cell Signaling Technology | 1:1000 for WB | Cat# 12721;  RRID: AB_2715528 |
| Rabbit anti-human METTL14 | Cell Signaling Technology | 1:1000 for WB | Cat# 51104;  RRID: AB_2799383 |
| Rabbit anti-human WTAP | Cell Signaling Technology | 1:1000 for WB | Cat# 41934;  RRID: AB_2923053 |
| Rabbit anti-human H3K9me3 | Cell Signaling Technology | 1:1000 for WB | Cat# 13969;  RRID: AB_2923053 |
| Rabbit anti-human H3K27me3 | Cell Signaling Technology | 1:1000 for WB | Cat# 9733;  RRID: AB_2616029 |
| Rabbit anti-human H3 | Cell Signaling Technology | 1:1000 for WB | Cat# 4499;  RRID: AB_10544537 |
| Rabbit anti-human N6-methyladenosine (m6A) | ABclonal | 1:1000 for WB;  1:100 for IF;  1:1000 for Dot blot | Cat# A19841;  RRID: AB_2862753 |
| Rabbit anti-human TriMethyl-Histone H3-K4 mAb | ABclonal | 1:1000 for WB | Cat# A2357;  RRID: AB_2631278 |
| Rabbit anti-human 5-Methylcytosine (5mC) | ABclonal | 1:1000 for Dot blot | Cat#A20599;  RRID: AB_3075522 |
| Rabbit anti-human METTL3 | ABclonal | 1:1000 for WB | Cat# A8370;  RRID: AB_2770344 |
| Rabbit anti-human PHGDH | ABclonal | 1:1000 for WB | Cat#A10461;  RRID: AB_2758010 |
| Rabbit anti-human PSAT1 | ABclonal | 1:1000 for WB | Cat# A6707; RRID: AB_2767290 |
| Rabbit anti-human SHMT1 | ABclonal | 1:1000 for WB | Cat# A7727;  RRID: AB_2772214 |
| Rabbit anti-human SHMT2 | ABclonal | 1:1000 for WB | Cat# A1215;  RRID: AB_2759037 |
| Rabbit anti-human HMOX1 | ABclonal | 1:1000 for WB | Cat#A19062;  RRID:AB_2862555 |
| Rabbit anti-human MTHFD2 | ABclonal | 1:1000 for WB | Cat# A22653;  RRID: AB_3075523 |
| Rabbit anti-human PSPH | ABclonal | 1:1000 for WB | Cat# A7924;  RRID: AB_2771862 |
| anti-rabbit IgG HRP conjugated | Abcam | 1:1000 for WB | Cat# ab6721;  RRID: AB_955447 |
| anti- mouse IgG HRP conjugated | Abcam | 1:1000 for WB | Cat# ab6789;  RRID: AB_955439 |
| Anti-Rabbit CoraLite®488-Conjugated AffiniPure Goat IgG(H+L) | Proteintech | 1:100 for IF | Cat#SA00013-2;  RRID: AB_2797132 |
| Anti-Mouse Alexa Fluor 594-conjugated AffiniPure Goat IgG | Jackson | 1:100 for IF | Cat#115-585-003;  RRID: AB_2338871  115-585-146 |

**Table S7** The names of the cell lines sensitive and resistant to Lenvatinib

| LIMOR | Sensitive | | | | | Resistant | | | | | | | | | | | | | | | | | | | | | | |
| --- | --- | --- | --- | --- | --- | --- | --- | --- | --- | --- | --- | --- | --- | --- | --- | --- | --- | --- | --- | --- | --- | --- | --- | --- | --- | --- | --- | --- |
|  | SNU398 | CLC13 | Hep3B | Huh7 | CLC16 | SNU423 | CLC18 | CLC19 | CLC20 | CLC24 | CLC26 | CLC27 | CLC2 | CLC37 | CLC38 | CLC41 | CLC42 | CLC43 | CLC44 | CLC46 | CLC47 | CLC4 | CLC50 | CLC7 | Li7 | SNU368 | SNU475 | SNU878 |
| CTRP-CCLE | Sensitive | | | Resistant | | | | | | | | | | | | | | | | | | | | | | | | |
|  | HEP3B217_LIVER | HUH7_LIVER | PLCPRF5_LIVER | SNU761_LIVER | HEPG2_LIVER | HLF_LIVER | HUH1_LIVER | HUH6_LIVER | JHH1_LIVER | JHH2_LIVER | JHH4_LIVER | JHH5_LIVER | JHH6_LIVER | JHH7_LIVER | LI7_LIVER | NCIH684_LIVER | SKHEP1_LIVER | SNU182_LIVER | SNU387_LIVER | SNU423_LIVER | SNU449_LIVER | SNU475_LIVER | SNU398_LIVER | SNU878_LIVER | SNU886_LIVER |  |  |  |

**Key Reagent or S****ource**

| **Critical commercial assays** | **Manufacturer** | **Cat No.** |
| --- | --- | --- |
| Annexin V-PE/7-AAD Apoptosis Detection Kit | Vazyme | A211-02 |
| Lactic Acid assay kit | Nanjing Jiancheng Bioengineering Institute | A019-2-1 |
| MTT | Thermo Scientific | Cat# M6494 |
| Magna RIP RNA-Binding Protein Immunoprecipitation Kit | Merck Millipore | 17-700 |
| Methylated RNA Immunoprecipitation (MeRIP) Kit | BersinBio^TM^ | Bes5203-2 |
| TIANamp Genomic DNA Kit (TIANGEN Biotech, Beijing, China) | TIANGEN | Cat#4992199 |
| Dual-Luciferase® Reporter Assay System, Firefly, Renilla | Promega | Cat# E1910 |
| FastPure® Cell/Tissue Total RNA Isolation Kit V2 | Vazyme Biotech Co.,Ltd | RC112 |
| ClonExpress Ultra One Step Cloning Kit V2 | Vazyme Biotech Co.,Ltd | C116 |
| 2 x Phanta Max Master Mix (Dye Plus) | Vazyme Biotech Co.,Ltd | P515 |
| FastPure Blood/Cell/Tissue/Bacteria DNA lsolationMini Kit | Vazyme Biotech Co.,Ltd | DC112 |
| CCK-8 Cell Counting Kit | Vazyme Biotech Co.,Ltd | A311 |
| Pierce™ Magnetic RNA-Protein Pull-Down Kit | Thermo | 20164 |
| Biolinkedin®Protein A/G magnetic beads | LinkedIn Biotechnology Co., Ltd. | L1204 |
| GSH and GSSG Assay Kit | Biovision | K264 |
| NADP+/NADPH Assay Kit | Beyotime | S0179 |
| CellROX™ Green | Invitrogen™ | C10444 |
| S-Adenosylmethionine (SAM) ELISA Kit | CELL BIOLABS | MET-5152 |
| Aspartate aminotransferase Assay Kit | Nanjing Jiancheng Bioengineering Institute | C010-2-1 |
| Alanine aminotransferase Assay Kit | Nanjing Jiancheng Bioengineering Institute | C009-2-1 |
| Histostain™ - SP Kits | ZSGB-BIO | PV-9000 |
| DAB kit | ZSGB-BIO | ZLI-9019 |
| Pierce Rapid Gold BCA Protein Assay | Thermo Scientific, America | Cat#1863381 |

| **Chemicals** | **Source** | **Cat No.** |
| --- | --- | --- |
| Lenvatinib | Selleck | S1164 |
| 2-Deoxy-D-glucose | Selleck | S4701 |
| NCT-503 | Selleck | Cat#S8619 |
| Sodium L-lactate | Selleck | Cat#S6010 |
| L-Buthionine sulfoximine (BSO) | Selleck | Cat#S9728 |
| N-acetyl-L-cysteine (NAC) | Selleck | Cat#S1623 |
| Ademetionine (SAM) | Selleck | Cat#S5109 |
| 3-Deazaadenosine hydrochloride (DAA) | Selleck | Cat#S0787 |
| Lipofectamine™ 3000 | Thermo Fisher Scientific | Cat#L3000001 |
| InvitroRN™️ Reagent | InvivoGene Biotechnology, Suzhou, China | Cat# IVG1101-10 |
| Opti-MEM | Gibco | Cat# 31985070 |
| DZNep(3-deazaneplanocinA) | Selleck | Cat#S7102 |
| 5-Azacytidine | Selleck | Cat#S1782 |
| L-Serine | MedChemExpress | Cat#HY-N0650 |
| DiR | MedChemExpress | Cat#HY-D1048 |
| 3-MPA hydrochloride | MedChemExpress | Cat#HY-128923 |
| Actinomycin D | MedChemExpress | Cat#HY-17559 |
| L-Serine standards | Shanghai Yuanye Bio-Technology Co., Ltd | Cat#56-45-1 |
| Glycine standards | Shanghai Yuanye Bio-Technology Co., Ltd | Cat#56-40-6 |
| PEP | Shanghai Yuanye Bio-Technology Co., Ltd | Cat#10526-80-4 |
| D-(−)-3-Phosphoglyceric acid disodium salt | Shanghai Yuanye Bio-Technology Co., Ltd | Cat#80731-10-8 |
| 2-NBDG | Invitrogen™ | Cat#N13195 |
| Sodium citrate dihydrate | Shanghai Hushi Laboratorial Equipment Co., Ltd | Cat#6132-04-3 |
| Citric acid | Shanghai Hushi Laboratorial Equipment Co., Ltd | Cat#5949-29-1 |
| U-[13C]- D-GLUCOSE | Cambridge Isotope Laboratories | Cat#CLM-1396-1 |
| U-[13C]- pyruvate | Cambridge Isotope Laboratories | Cat#CLM-2440-0.1 |
| Penicillin-Streptomycin Solution | Meilunbio | Cat#MA0110 |
| DMEM (no glucose) | Gibco | Cat#A1443001 |
| MEM (no glucose) | Procell | Cat#PM150445 |
